# Recyclable optical bioplastics platform for solid state red light harvesting *via* triplet–triplet annihilation photon upconversion†

**DOI:** 10.1039/d2ta04810h

**Published:** 2022-08-16

**Authors:** Pankaj Bharmoria, Fredrik Edhborg, Hakan Bildirir, Yoichi Sasaki, Shima Ghasemi, Anders Mårtensson, Nobuhiro Yanai, Nobuo Kimizuka, Bo Albinsson, Karl Börjesson, Kasper Moth-Poulsen

**Affiliations:** Department of Chemistry and Chemical Engineering, Chalmers University of Technology Kemivägen 4 Gothenburg 412 96 Sweden pankajb@chalmers.se kasper.moth-poulsen@chalmers.se; Department of Applied Chemistry, Graduate School of Engineering, Center for Molecular Systems (CMS), Kyushu University 744 Moto-oka, Nishi-ku Fukuoka 819-0395 Japan; Department of Chemistry and Molecular Biology Kemivägen 10 Gothenburg 412 96 Sweden; The Institute of Materials Science of Barcelona, ICMAB-CSIC Bellaterra Barcelona, 08193 Spain; Catalan Institution for Research & Advanced Studies, ICREA Pg. Lluís Companys 23 Barcelona Spain

## Abstract

Sustainable photonics applications of solid-state triplet–triplet annihilation photon upconversion (TTA-UC) are limited by a small UC spectral window, low UC efficiency in air, and non-recyclability of polymeric materials used. In a step to overcome these issues, we have developed new recyclable TTA-UC bioplastics by encapsulating TTA-UC chromophores liquid inside the semicrystalline gelatin films showing broad-spectrum upconversion (red/far-red to blue) with high UC efficiency in air. For this, we synthesized a new anionic annihilator, sodium-TIPS-anthracene-2-sulfonate (TIPS-AnS), that combined with red/far-red sensitizers (PdTPBP/Os(*m*-peptpy)_2_(TFSI)_2_), a liquid surfactant Triton X-100 reduced (TXr) and protein gelatin (G) formed red/far-red to blue TTA-UC bioplastic films just by air drying of their aqueous solutions. The G-TXr-TIPS-AnS-PdTPBP film showed record red to blue (633 to 478 nm) TTA-UC quantum yield of 8.5% in air. The high UC quantum yield has been obtained due to the fluidity of dispersed TXr containing chromophores and oxygen blockage by gelatin fibers that allowed efficient diffusion of triplet excited chromophores. Further, the G-TXr-TIPS-AnS-Os(*m*-peptpy)_2_(TFSI)_2_ bioplastic film displayed far-red to blue (700–730 nm to 478 nm) TTA-UC, demonstrating broad-spectrum photon harvesting. Finally, we demonstrated the recycling of G-TXr-TIPS-AnS-PdTPBP bioplastics by developing a downstream approach that gives new directions for designing future recyclable photonics bioplastic materials.

## Abstract 2

Sustainable red light harvesting has been achieved in recyclable protein–surfactant–chromophores co-assembled bioplastics with high efficiency due to the efficient diffusion of triplet excited chromophores, and oxygen blockage by protein fibers.

## Graphical abstract

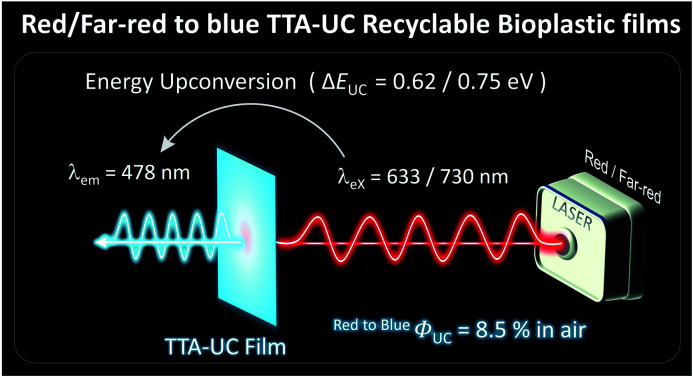

## Introduction

Photon upconversion (PUC) is a process of transforming low-energy photons into high-energy photons.^1–3^ Among the known PUC processes,^1–8^ triplet–triplet annihilation photon upconversion (TTA-UC) has an advantage over energy transfer upconversion (ETU), excited-state absorption (ESA), and photon avalanche (PA), due to the operation at flexible spectral ranges, and at low excitation intensities.^9–11^ TTA-UC occurs in an ensemble of chromophores, wherein a sensitizer after absorbing low energy generates triplet states, followed by triplet energy transfer (TET) to the annihilator *via* a Dexter energy transfer mechanism. The annihilator triplets then undergo TTA to form an emissive singlet state, which radiates the anti-Stokes delayed fluorescence (Fig. S1†).^12,13^ Due to the high UC quantum yields at low excitation intensities,^14–16^ TTA-UC has attracted a plethora of applications not limited to photocatalysis,^17^ biological imaging,^18^ 3-D printing^19^ and photovoltaics.^20,21^

In photovoltaics, TTA-UC can increase the efficiency of solar cells, by upconverting the otherwise transmitted sub-band gap photons to photons corresponding to the bandgap. But, practical integration with photovoltaics requires efficient solid-state TTA-UC materials with a broad UC spectral window (Red/NIR to vis).^22,23^ However, realizing efficient solid-state TTA-UC materials face several challenges like aggregation-induced emission quenching, back energy transfer, triplet quenching by molecular oxygen, and lack of sensitizer–annihilator pairs with suitable triplet energies.^11,24,25^ These challenges have been addressed partly in many proofs-of-concept solid-state green to blue TTA-UC systems.^24–26^ However, practical applications demand an extension of the photon harvesting window to red or far-red/near-infrared photons (far-red/NIR), which is the key goal of this work.^12,20–23^ Existing literature on solid-state red to blue molecular TTA-UC is limited to examples of chromophore doped synthetic polymeric films of polyurethanes,^27,28^ methyl acrylate,^29^ TTA-UC crystals (*Φ*_UC_ = 5.6%),^30^ and chromophores loaded into liquid nanocapsules dispersed in cross-linked cellulose nanofiber films with *Φ*_UC_ = 8.2% in deaerated conditions.^31^ Regarding solid-state near-infrared/far-red to visible (NIR/far-red to vis) molecular TTA-UC, different approaches to upconvert NIR/far-red light to red, yellow, green, blue, and violet light in PVA-doped chromophore nanoparticles,^32–34^ metal–organic frameworks^35^ and crystals,^36^ amorphous solid microparticles,^37^ and chromophores doped polystyrene film have been reported.^38^

However, low *Φ*_UC_ in air and non-recyclable petroleum-derived plastics used in these systems remains key practical challenges for their sustainable applications. Many petroleum based-plastics are non-biodegradable which alarmingly leads to interference with the ecological and food cycles in the form of bulk plastic waste and/or microplastics.^39,40^ Moreover, it might be hard to recycle chromophores used in such plastics for photonics applications. Therefore, it is of great importance to develop techniques for plastic^41^ and dye recycling or explore the potential use of biopolymers^42–45^ as an alternative for solid-state TTA-UC. To address this issue, we previously developed a proof-of-concept photon upconversion bioplastic approach for green to blue TTA-UC with *Φ*_UC_ = 7.8% in air.^46^ However, a limited UC spectral window remains an issue, due to the lack of ionic annihilators with suitable triplet energies to couple with red/far-red/NIR sensitizers, which is a key requisite for fabricating optically active TTA-UC bioplastics.

**Fig. 1:**
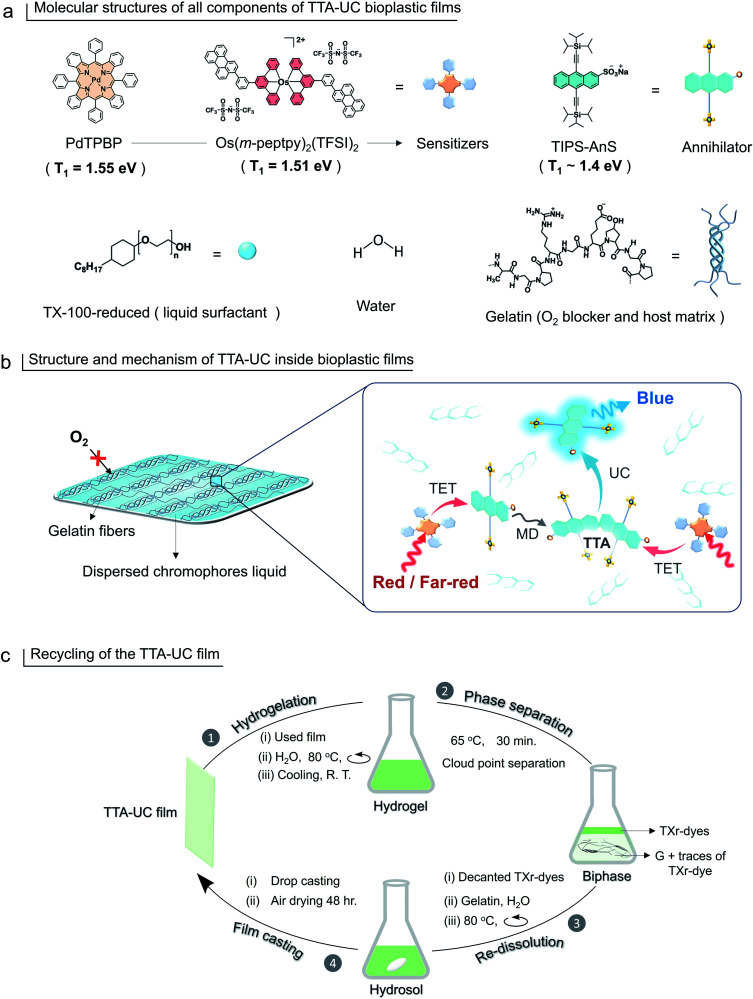
(a) Molecular structures of sensitizers (PdTPBP and Os(*m*-peptpy)_2_(TFSI)_2_) and annihilator (TIPS-AnS), TXr, water, and gelatin. (b) Structure of an aqueous processed G-TXr-TIPS-AnS-PdTPBP or G-TXr-TIPS-AnS-Os(*m*-peptpy)_2_(TFSI)_2_ films showing dispersed TXr-chromophores liquid trapped inside the gelatin fibers acting as an oxygen blocker and TTA-UC mechanism inside the dispersed TXr-chromophores liquid. (c) Illustration of the recycling of TTA-UC bioplastic film *via* extraction of TXr-dyes from gelatin through phase separation at the cloud point of TXr followed by redissolution in fresh gelatin and film casting.

In this work we have addressed this challenge by synthesizing a new ionic annihilator; sodium TIPS-anthracene 2-sulfonate (TIPS-AnS). TIPS-AnS has a suitable triplet energy (*T*_1_ ∼ 1.41 eV^47,48^) to pair with a red sensitizer, Pd(ii) *meso*-tetraphenyl tetrabenzoporphine (PdTPBP, *T*_1_ = 1.55 eV).^49,50^ Hence, the prepared gelatin-TX-100-reduced-TIPS-AnS-PdTPBP (G-TXr-TIPS-AnS-PdTPBP) bioplastic film showed an efficient red-to-blue TTA-UC (anti-stokes shift, Δ*E* = 0.62 eV), with a high UC quantum yield, *Φ*_UC_ = 8.5% (50% theoretical maximum) and a reasonably low excitation intensity of *I*_th_ = 95 mW cm^−2^. This is a record *Φ*_UC_ for solid-state red to blue TTA-UC in air. The UC spectral range was further expanded to harvest far-red photons (700–730 nm) by pairing TIPS-AnS with a far-red/NIR sensitizer, Os(*m*-peptpy)_2_(TFSI)_2_ (*T*_1_ = 1.51 eV^51^) to form G-TXr-Os(*m*-peptpy)_2_(TFSI)_2_-TIPS-AnS, far-red to blue TTA-UC bioplastic film. Hence, compared to our previous report^46^ we have formulated new sensitizer–annihilator pairs with suitable triplet energies that expand the UC spectral window to the far-red region. Further, we replaced the toxic TX-100 (currently being phased out in Europe due to legislation) with the globally acceptable TX-100-reduced (TXr)^52^ to dissolve chromophores in the film. Interestingly, this small change in chemical structure drastically changed the film nanostructure wherein TXr was found as a continuously dispersed liquid unlike the isolated droplets of TX-100 in the gelatin film reported by us previously.^46^ Finally, to avoid the post-utilization waste of developed bioplastics, a proof-of-concept downstream recycling approach of “cloud point extraction of TXr-chromophores” from the used G-TXr-TIPS-AnS-PdTPBP film has been developed. The extracted TXr-chromophores were reused to fabricate a new TTA-UC film. Hence, the highly efficient and recyclable TTA-UC bioplastics developed in this work convey a new direction to the solid-state TTA-UC field for sustainable broad-spectrum photon harvesting. The molecular structure of gelatin, TXr, PdTPBP, Os(*m*-peptpy)_2_(TFSI)_2_, and TIPS-AnS are shown in Fig. 1a, and a pictorial presentation of the mechanism of TTA-UC in the films, and recycling are shown in Fig. 1b and c.

## Experimental section

### Materials

All solvents and reagents were used as received. All solvents used in this work were purchased from Fischer Scientific. Palladium(ii) *meso*-tetraphenyl porphine (PdTPBP) was purchased from Frontier Scientific. TX-100-reduced, sodium anthraquinone-2-sulfonate, (triisopropylsilyl)acetylene (97%), tin(ii) chloride reagent grade (98%), gelatin type A porcine skin (80 to 120 g bloom) were purchased from Sigma Aldrich. The Os(*m*-peptpy)_2_(TFSI)_2_ was synthesized in our lab (Kimizuka Lab, Kyushu University, Japan). The synthesis and characterization of this sensitizer is published recently elsewhere.^51^ TIPS-anthracene-2-sulfonate was synthesized using the procedure given below.

### Synthesis and purification of sodium TIPS-anthracene 2-sulfonate

The synthetic procedure was adapted from the literature on the synthesis of non-sulfonated (triisopropylsilyl)acetylene-anthracene compounds.^53,54^ The two-step reaction involves the addition of lithium (triisopropylsilyl)acetylide to the carbonyls of sodium anthraquinone-2-sulfonate and the following elimination *via* SnCl_2_ to form the targeted anthracene derivative. In detail; 1.6 mL of (triisopropylsilyl)acetylene (TIPS) was dissolved in 20 mL anhydrous THF under a nitrogen atmosphere and cooled to −78 °C. 4.4 mL *n*-butyl lithium (1.6 M in hexane) was added to the solution dropwise, and the mixture was further stirred at −78 °C for 1 h. In the following, the prepared Li-TIPS salt solution was heated to room temperature for a few minutes (5–7 min) and transferred (*via* a cannula) to a flask containing the solution of dried (to get rid of the adsorbed water) 1 g sodium anthraquinone-2-sulfonate in 20 mL anhydrous THF at −78 °C under nitrogen atmosphere. The pale-yellow mixture was stirred at −78 °C for 30 min. and heated to room temperature for an overnight stirring (*ca.* 15 h). The final orange suspension was quenched with 5 mL of water, and the solid was filtrated using a Büchner funnel and flask. The filtrate was dissolved in diethyl ether and extracted with water. The collected organic phase was dried using sodium sulfate, filtrated, and the solvent evaporated to obtain a crude orange solid to be processed in the next step. The crude orange solid was dissolved in 5 mL THF and slowly added to the flask containing the 2 g SnCl_2_ in 10 mL THF. The mixture turned to a thick dark red solution, which was diluted using 10 mL THF and heated to 45 °C to be stirred overnight. The mixture was then quenched with 20 mL water and extracted using an excess of diethyl ether 3 times. The organic phase was dried by using sodium sulfate, and the solvent of the separated organic solution was removed under vacuum. The brown solid was washed with EtOH and purified *via* a silica gel column (ethyl acetate : methanol/99 : 1). The collected compound was then recrystallized in MeOH, yielding a 5% overall reaction. The yellow precipitates of the compound obtained after drying were checked for ^1^H-NMR, ^13^C-NMR, and mass analysis to ascertain the purity (Fig. S2–S4†).

^1^H (d-THF, 400 MHz) *δ* 9.25 (s, 1H), 8.68–8.60 (m, 2H), 8.52 (d, 1H), 8.20 (d, 1H), 7.63 (dd, 2H), 1.3–0.8 (m, 42H).

^13^C (d-THF & CDCl_3_ mix, 100 MHz) *δ* 33.22, 132.19, 131.94, 131.73, 130.68, 128.81, 126.66, 126.49, 126.46, 126.39, 124.46, 124.34, 119.35, 117.68; 105.02, 104.10, 102.45, 102.11; 17.90, 10.78.

*m*/*z* [M^+^H]^+^ calculated 641.2911, found 641.2917.

### Sample preparation

#### Preparation of G-TXr-TIPS-AnS-PdTPBP film

For the preparation of G-TXr-TIPS-AnS-PdTPBP films (sample-I, sample-II, and sample-III) 250 μl, 125 μl, and 62.5 μl stock solutions of TIPS-An in methanol (4 mM) were taken in three different glass vials and evaporated under reduced pressure. To the dried TIPS-AnS residues added 0.026 g of TX-100-reduced containing PdTPBP (25 μM), followed by stirring at 80 °C for complete mixing. To the resulting solution added 1 mL of water, followed by 0.22 g of gelatin (G). The G-TXr-TIPS-AnS-PdTPBP solution was stirred at 80 °C for 10 min. The hot sol was allowed to rest at room temperature for 2 minutes, followed by drop-casting of the 260 μl of the sol on a 3 × 1 cm glass plate, followed by air drying for 48 h. The dried films contain similar concentrations of TXr (10.5%), gelatin (89.4%), and PdTPBP (27 μmol kg^−1^), whereas the concentrations of TIPS-AnS were varied between 5, 2.5, and 1 mmol kg^−1^ respectively for sample-I, sample-II, and sample-III. The real-time schematic of the G-TXr-TIPS-AnS-PdTPBP film (sample-I) preparation is shown in Fig. S5.† The semi-transparent film obtained after air drying for 48 h is shown in Fig. S5,† and Fig. 3a of the main manuscript.

**Fig. 2:**
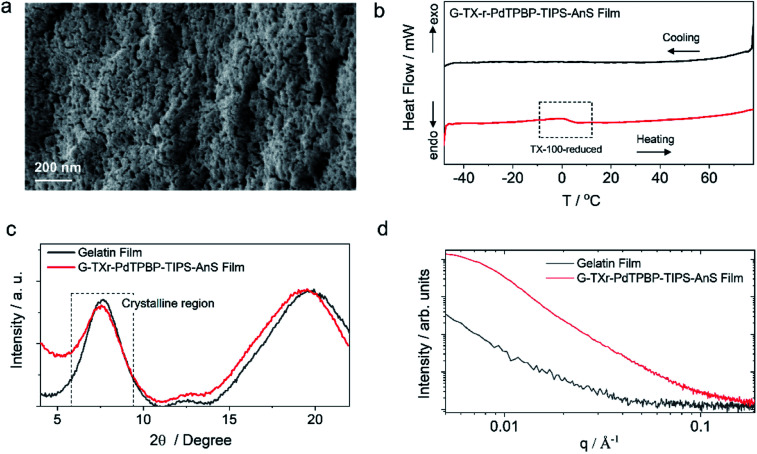
Structural characterization of G-TXr-PdTPBP-TIPS-AnS film. (a) Cross-section SEM image showing the thick fiber network structure of the core of the film. (b) DSC thermogram showing glass transition at the melting temperature of TX-100-reduced inside the film. (c) WAXS pattern showing the semicrystalline nature of the film. (d) Transmission mode SAXS pattern.

**Fig. 3:**
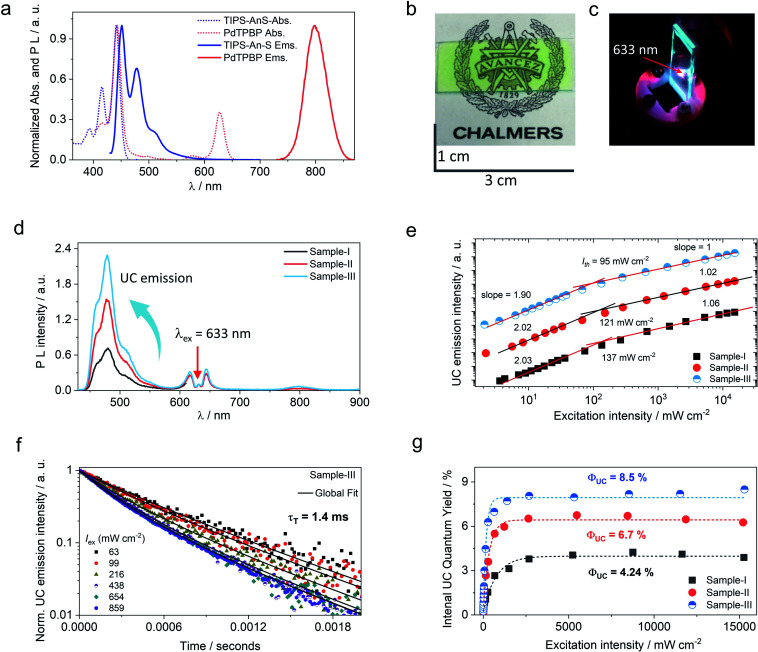
Optical characterization of G-TXr-TIPS-AnS-PdTPBP film. (a) Absorption (dotted lines) and PL (solid lines) spectra of the G-TXr-TIPS-AnS (blue line, *λ*_ex_ = 415 nm, TIPS-AnS = 200 μmol kg^−1^) and G-TXr-PdTPBP (red line, *λ*_ex_ = 633 nm, 27 μmol kg^−1^) films in the air. (b) Photograph of G-TXr-TIPS-AnS-PdTPBP film under white light, and (c) under 633 nm laser excitation in the air (excitation intensity = 1.3 W cm^−2^, no short pass filter used). (d) Comparative PL spectra of G-TXr-PdTPBP-TIPS-AnS films at different concentrations of TIPS-AnS (1, 2.5 and 5 mmol kg^−1^) upon 633 nm laser excitation (excitation intensity = 33.8 mW cm^−2^). (e) Comparative log–log plot of excitation intensity dependence of UC emission intensity of G-TXr-TIPS-AnS-PdTPBP films at different concentrations of TIPS-AnS. (f) Time traces of normalized upconversion emission intensity of G-TXr-TIPS-AnS-PdTPBP sample-III for various excitation intensities in the air (*λ*_ex_ = 633 nm laser, *λ*_em_ = 478 nm). (g) Comparative internal UC quantum yield of G-TXr-TIPS-AnS-PdTPBP films in the air.

#### Preparation procedure for G-TXr-TIPS-AnS-Os(*m*-peptpy)_2_(TFSI)_2_ film

**Fig. 4:**
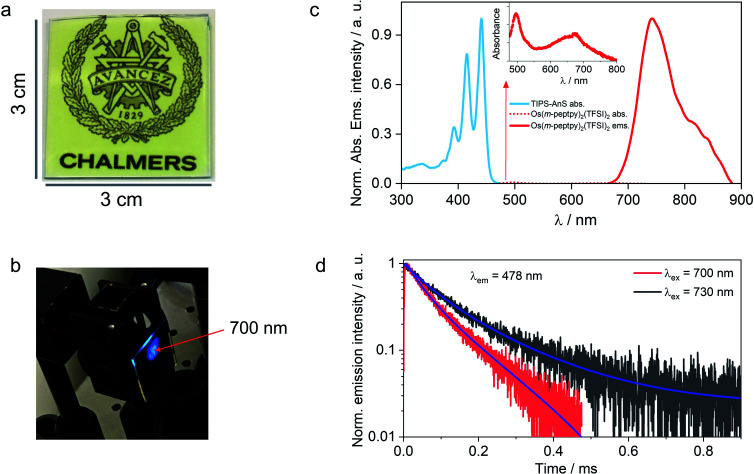
Far-red to blue TTA-UC bioplastic film in air. (a) and (b) Photographs of G-TXr-TIPS-AnS-Os(*m*-peptpy)_2_(TFSI)_2_ film under white light and under 700 nm nanosecond pulsed laser excitation in the air with excitation intensity = 0.9 mJ pulse^−1^ (no short pass filter used). (c) Normalized absorption, and emission spectra of G-TXr-TIPS-AnS-Os(*m*-peptpy)_2_(TFSI)_2_ film wherein the blue line shows the absorption spectrum of TIPS-AnS, and the red lines show absorption (inset, dotted line) and emission (solid line, *λ*_ex_ = 640 nm) spectra of Os(*m*-peptpy)_2_(TFSI)_2_ in the film. (d) UC emission rise and decay profile of G-TXr-TIPS-AnS-Os(*m*-peptpy)_2_(TFSI)_2_ film in the air upon 700 nm and 730 nm nanosecond pulsed laser excitation.

For the preparation of G-TXr-TIPS-AnS-Os(*m*-peptpy)_2_(TFSI)_2_ film, 250 μl of the stock solution of TIPS-An in methanol (4 mM) was taken in a glass vial, followed by methanol evaporation under reduced pressure. To the solid TIPS-AnS residue added 62.5 μl of Os(*m*-peptpy)_2_(TFSI)_2_ solution in DMF (200 μM), followed by DMF evaporation under reduced pressure. To the resulting TIPS-AnS-Os(*m*-peptpy)_2_(TFSI)_2_ residue added 0.026 g of TX-100-reduced followed by stirring at 80 °C. To the TIPS-AnS-Os(*m*-peptpy)_2_(TFSI)_2_ solution added 1 mL of water followed by 0.22 g of gelatin and stirring for 10 min. at 80 °C. The hot sol obtained after stirring was allowed to rest at room temperature for 2 min. followed by casting of the 500 μl of the sol on a 3 × 3 cm glass plate and air drying for 48 h. The transparent yellow film obtained after 48 h of air drying is shown in the Fig. 4a of the main manuscript. The dried film contains TXr (10.5%), gelatin (89.4%), Os(*m*-peptpy)_2_(TFSI)_2_ (63 μmol kg^−1^) and TIPS-AnS (5 mmol kg^−1^).

#### Preparation of G-TXr-TIPS-AnS-cresyl violet perchlorate films

250 μl, 125 μl, and 62.5 μl stock solution of TIPS-An in methanol (4 mM) taken in three different glass vials was evaporated under reduced pressure. To this added 22 μl of cresyl violet perchlorate solution in ethanol (553 μM) and evaporated under reduced pressure. To the resulting residue, added 0.026 g of TX-100-reduced and stirred at 80 °C for mixing the dyes with TXr. To this added 1 mL of water, followed by stirring and further addition of 0.22 g of gelatin and stirring for 10 min at 80 °C. G-TXr-TIPS-AnS-cresyl violet perchlorate hot sol was then allowed to rest at room temperature for 2 min. followed by casting 260 μl of the sol on a 3 × 1 glass plate. The casted solution was then allowed to dry at room temperature for 48 h in the air resulting in the formation of G-TXr-TIPS-AnS-cresyl violet perchlorate films. The concentration of cresyl violet perchlorate in the films is 50 μM.

### Measurements

#### Optical measurements

All UV-vis absorption spectra were recorded on a Varian Cary 50 spectrophotometers. Steady-state emission spectra were recorded on a Varian Eclipse spectrophotometer. The UV-vis and emission spectra in solution were recorded using 2 mm pathlength quartz cuvette. The steady-state photon upconversion emission measurements were recorded using a home-built setup consisting of Coherent OBIS LS 633 nm diode laser as the excitation source, a 1681 SPEX monochromator, and a photomultiplier tube (PMT) detector. A 633 nm notch filter was used in front of the monochromator to reduce the scattered excitation light reaching the detector. The UC emission measurement in the TXr-TIPS-AnS-PdTPBP degassed solution was carried out in the 1 cm pathlength quartz cuvette. The fluorescence lifetime of TIPS-AnS in the films and TXr liquid were measured using time-correlated single-photon counting (TCSPC) with a 405 nm laser diode (PicoQuant) as the excitation source and a microchannel plate-PMT (MCP-PMT) detector in an Edinburgh Instruments LifeSpec II. The measurement in the TXr was carried out in a 2 mm pathlength quartz cuvette. Upconversion kinetics in G-TXr-TIPS-AnS-PdTPBP films was investigated by ns time-resolved emission spectroscopy using a home-built system. The excitation source was a Coherent OBIS LS 633 nm continuous-wave laser coupled to a pulse generator. The pulse generator was used to control the excitation pulse width, which was set to 25 ms. An Oriel Cornerstone 130 monochromator was used in front of a five-stage PMT detector. A 633 nm notch filter was used in front of the monochromator to reduce the scattered excitation light reaching the detector. Upconversion kinetics in G-TXr-TIPS-AnS-Os(*m*-peptpy)_2_(TFSI)_2_ film using the 700 and 730 nm excitation was investigated using Spectra-Physics Quanta-Ray Nd:YAG laser with a Primoscan OPO. All ns time-resolved emission measurements were carried out with an excitation laser beam at a right angle to the line of detection and the films at approximately 30° to the direction of the excitation light to yield front-face detection. The absolute fluorescence quantum yields of G-TXr-TIPS-AnS-cresyl violet (*λ*_ex_ = 633 nm), and G-TXr-TIPS-AnS-PdTPBP (*λ*_ex_ = 415 nm) films were measured in the integrated sphere using FLS1000 Photoluminescence Spectrometer, Edinburgh instrument. The measurements were carried out by putting sample or reference films cast on a glass plate horizontally on a sample stage, followed by 90° excitation with respect to the detector. Before the sample measurements, photoluminescence spectra (PL) of empty glass plates were recorded as reference. For G-TXr-TIPS-AnS-cresyl violet film the PL spectra were recorded between 613 nm to 800 nm (*λ*_ex_ = 633 nm). The absolute quantum was calculated using instrument inbuilt software by subtracting the reference spectrum between 640 to 800 nm. The absolute quantum yield value of cresyl violet was calculated to be 0.31. The data wasn't corrected for reabsorption. The same wavelength range of cresyl violet's PL spectra in the G-TXr-TIPS-AnS-cresyl violet film measured at different excitation intensities of 633 nm laser was used for relative UC quantum yield measurement. For G-TXr-TIPS-AnS-PdTPBP film the PL spectra were recorded between 395 nm to 650 nm (*λ*_ex_ = 415 nm). The Q. yield was measured between 420 to 650 nm. The samples were not corrected for reabsorption. The phosphorescence lifetime was measured using Varian Eclipse 1 fluorescence spectrophotometer with 633 nm excitation.

#### Determination of relative fluorescence quantum yield of TIPS-AnS in TX-100 reduced

The relative fluorescence quantum yield of TIPS-AnS in TXr liquid was measured using coumarin 153 in ethanol in a quartz cuvette (pathlength = 2 mm), as a reference standard using eqn (1). The quantum yield of coumarin 153 in ethanol used for the calculation is 0.537. This value is calculated at Chalmers University of Technology, Sweden, and is comparable to the literature value.^55^1
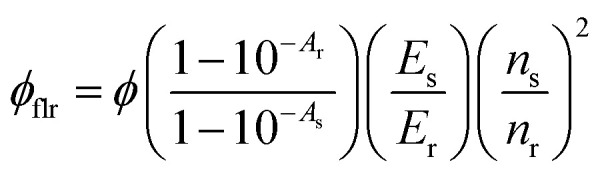
Where *ϕ*_flr_, *ϕ*_r_, *A*_s_, *A*_r_, *E*_s_, *E*_r_ and *n*_s_, *n*_r_ are quantum yield, absorbance, integrated emission profiles, and refractive index of sample and reference standard, respectively. To avoid the inner filter effect on the quantum yield measurement, absorbance of both sample and reference samples were maintained below 0.1 during the measurements.

#### Determination of relative upconversion quantum yields in the G-TXr-TIPS-AnS-PdTPBP films

The relative upconversion quantum yield of G-TXr-TIPS-AnS-PdTPBP films was measured using Cresyl violet perchlorate as a reference standard in G-TXr-TIPS-AnS-Cresyl violet film of the same thickness due to the overlap of its absorption spectrum with PdTPBP using eqn (4).2
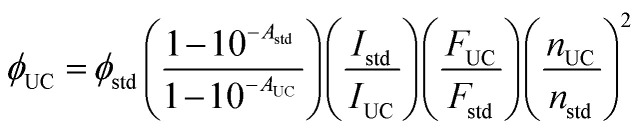
Where *ϕ*_UC_, *ϕ*_std_, *A*_UC_, *A*_std_, *I*_UC_, *I*_std_, *F*_UC_, *F*_std_ and *n*_UC_, *n*_std_ are quantum yield, absorbance, excitation intensity, integrated UC emission profiles, and refractive index of the UC sample and reference standard, respectively. The average absolute quantum yields of cresyl violet perchlorate in the G-TXr-TIPS-AnS-Cresyl violet films measured using an integrated sphere was found to be 0.31. This value was used for the calculation of UC quantum yields in the TTA-UC films. The same thickness of the sample and reference films were maintained by casting an equal amount of solution on the same size glass plate (3 × 1 cm). The UC emission spectra of G-TXr-TIPS-AnS-PdTPBP film showed an emission maximum at 478 nm due to the reabsorption of the original emission maximum at 451 nm caused by the secondary inner filter effect. Therefore, we calculated the internal UC quantum yield of the films by correcting for the secondary inner filter effect using a conversion factor of 0.016. The conversion factor was calculated using normalized emission spectra of TIPS-AnS in TXr at a low concentration (40 μM) with no secondary inner filter effect (Fig. S6†) using eqn (3).3
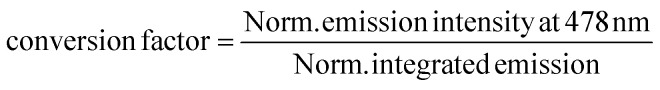


The obtained value was used to obtain to calculate integrated emission intensity without reabsorption using 0.016 as a conversion factor.

#### Other measurements

The film thickness was measured using a vernier caliper, Fowler, no. 659064. The average thickness of the film was found to be 0.13 mm. Wide-angle X-ray scattering and small-angle X-ray scattering of the G-TXr-TIPS-AnS-PdTPBP film were recorded in transmission mode using Mat: Nordic SAXS/WAXS/GISAXS instrument under vacuum. Differential scanning calorimetry (DSC) traces were obtained by using a TGA/DSC 3^+^ STAR^e^ system (METTLER TOLEDO) under an N_2_ atmosphere. Before performing measurements, samples were completely dried under vacuum for at least 24 h. The scanning rate was 10 °C min^−1^. After the first cycle, both film and TX-100 samples were kept at the isothermal condition at 100 °C before any further measurement. Reproducible thermograms of the 5th and 6th cycles of both the samples were considered for presentation. The cross-section Scanning electron microscopy (SEM) images of the G-TX-PtOEP-DPAS film were obtained using JEOL JSM-7800F Prime FEG SEM. The acceleration voltage was set to 10 kV and a secondary electron detector was used. Before SEM observation, the sample was dried under vacuum for two days and coated with gold using Edwards S150B gold sputter. The cross-section was obtained by breaking the film in half with hands. DMA analysis was performed on TA Instrument DMAQ800 in tensile film mode at a strain of 0.05% and frequency of 1 Hz and temperature of 30 °C. Prior to the measurement, a strain sweep was performed on G film to ensure that the applied strain is within the linear viscoelastic region.

## Results and discussion

### Synthesis and characterization of sodium TIPS-anthracene-2-sulfonate

TIPS-AnS was synthesized in a two-step synthesis involving the addition of lithium (triisopropylsilyl)acetylide to the carbonyls of sodium anthraquinone-2-sulfonate and the following elimination *via* SnCl_2_ to form the targeted anthracene derivative (see methods for detailed synthesis procedure). The purity of TIPS-AnS was confirmed from ^1^H-NMR, ^13^C-NMR, and HR-MS analysis (Fig. S2–S4†). The photophysical characterization of TIPS-AnS was carried out in THF and TXr. The absorption spectrum of TIPS-AnS in THF showed vibronic peaks at 392, 415, and 440 nm, whereas the emission maximum was observed at 449 nm, followed by peaks at 476 nm and 506 nm (Fig. S7†). In TXr the TIPS-AnS showed absorption at 394, 416, and 441 nm, and emission at 452, 478, and 510 nm. (Fig. S8†). Compared to the non-sulfonated TIPS-anthracene in cyclohexane, the absorption and emission spectra of TIPS-AnS in THF are red-shifted by 1 nm and 7 nm respectively (Fig. S9†).^47,48^ The fluorescence lifetime (*τ*_f_) and fluorescence quantum yield (*Φ*_f_) of TIPS-AnS in TXr were found to be, *τ*_f_ = 7.1 ns (Fig. S10†) and *Φ*_f_ = 89%. The TIPS-anthracene has a suitable triplet energy (*T*_1_ ∼ 1.4 eV)^47^ for exergonic triplet sensitization by PdTPBP (*T*_1_ = 1.55 eV,^49,50^ Fig. S8†) and Os(*m*-peptpy)_2_(TFSI)_2_ (*T*_1_ = 1.65 eV^51^). Therefore, we paired TIPS-AnS with PdTPBP and Os(*m*-peptpy)_2_(TFSI)_2_ to fabricate a G-TXr bioplastics film to investigate solid-state red/far-red to blue TTA-UC.

### Preparation and structural and optical characterization of G-TXr-TIPS-AnS-PdTPBP films

G-TXr-TIPS-AnS-PdTPBP films were prepared by drop-casting of their hot aqueous solutions on glass plates, followed by air drying for 48 h (see methods section and Fig. S5†). The semi-transparent films were obtained after drying due to the inter-chain cross-linking of gelatin upon dehydration, which resulted in an oxygen impermeable three-dimensional thick fiber network.^56^ Three different films were prepared at different TIPS-AnS concentrations (5, 2.5, and 1 mmol kg^−1^), and keeping the same concentration of TXr (10.5%), gelatin (89.4%), and PdTPBP (27 μmol kg^−1^). The transparency of G-TXr-TIPS-AnS-PdTPBP films increases with an increase in the concentration of anionic TIPS-AnS which is a characteristic feature of these biopolymer-surfactant-chromophores co-assembled molecular systems.^46,57^ Before, TIPS-AnS, we also tried non-ionic TIPS-anthracene, and tetratert-butylperylene, to pair with PdTPBP or Os(*m*-peptpy)_2_(TFSI)_2_ to fabricate bioplastics. However, these films are quite scattering and not suitable for correct TTA-UC characterization. Hence, justify the need for annihilators with ionicity or other designs suitable for co-assembly with the protein-surfactant system to fabricate such bioplastics. As per TIPS-AnS concentration, the prepared films were denoted as sample-I (TIPS-AnS = 5 mmol kg^−1^), sample-II (TIPS-AnS = 2.5 mmol kg^−1^), and sample-III (TIPS-AnS = 1 mmol kg^−1^) in the manuscript and ESI.† The variation in TIPS-AnS concentrations from sample-I to sample-III can be seen from their comparative absorption spectra (Fig. S11†).

Sample-I was used for structural characterization. A cross-section SEM image of sample-I shows a porous structure with thick fiber networks (Fig. 2a). It is different from the G-TX-DPAS-PtOEP film published previously by us;^46^ wherein liquid droplets of TX-100 surrounded by gelatin fibers were observed. Hence, in these films (sample-I to III) TXr could be present as a dispersed liquid rather than isolated droplets. This observation was further supported by temperature-dependent differential scanning calorimetry (DSC) thermograms of sample-I wherein a glass transition (Fig. 2b) was observed near the melting temperature of TXr at around 3 °C (Fig. S12†). It is different from the sharp endothermic peak observed for isolated TX-100 droplets in the G-TX-DPAS-PtOEP film published previously.^46^ The semicrystalline structure of the G-TXr-TIPS-AnS-PdTPBP film was confirmed from the broad peak at 2*θ* = 7.6° in the wide-angle X-ray scattering (WAXS) pattern (Fig. 2c) corresponding to the crystalline triple helices of gelatin with an inter-helix distance of 1.2 nm.^46,58^ The higher scattering of X-ray at small-angle (SAXS) in G-TXr-TIPS-AnS-PdTPBP film compared to gelatin film confirmed densely ordered nanostructure of dispersed TXr-TIPS-AnS-PdTPBP phase in the gelatin (Fig. 2d).^59^ The time sweep dynamic mechanical analysis of the film confirmed good mechanical stability with a constant storage modulus of 2300 MPa. (Fig. S13†).^46^

The molecular dispersion of chromophores (PdTPBP and TIPS-AnS) in the G-TXr film was confirmed by measuring their separate absorption and photoluminescence (PL) spectra (Fig. 3a). The absorption spectra of the G-TXr-TIPS-AnS film showed vibronic peaks at 393, 416, and 441 nm, whereas the emission maximum was observed at 451 nm, followed by peaks at 479 nm and 510 nm. Absorption spectra of the G-TXr-PdTPBP film showed a characteristic Soret band at 443 nm and Q bands at 581 and 628 nm, whereas the phosphorescence maximum was observed at 798 nm (1.55 eV).^49,50^ The similarity of these spectra with the absorption/emission peaks of TIPS-AnS and PdTPBP in the native TXr liquid (Fig. S8†) indicates their molecular dispersion in these films. However, TIPS-AnS showed slightly lower *Φ*_f_ = 79% and *τ*_f_ = 5.4 ns (Fig. S14,† violet line) in the G-TXr-TIPS-AnS film (TIPS-AnS = 200 μmol kg^−1^) compared to that in native TXr liquid (*Φ*_f_ = 89% and *τ*_f_ = 7.1 ns). The concentration-dependent emission decay profiles of TIPS-AnS in the presence of sensitizer in sample-I to sample-III showed a further decrease in *τ*_f_ to around 4.4 ns (Fig. S14†). These changes in *τ*_f_ can be assigned to the change in the chromophore's environment and structure of the film.^60^ We also observed the concentration-dependent secondary inner filter effect of TIPS-AnS in G-TXr-TIPS-AnS-PdTPBP films, from the decrease in its emission maximum at 451 nm (Fig. S15†).^61^ No change in emission decay profiles of TIPS-AnS in sample-I to sample-III with and without polarizer discards any fluorescence anisotropic effects.

### TTA-UC measurements of G-TXr-TIPS-AnS-PdTPBP films

Upon continuous excitation with a 633 nm laser, the G-TXr-TIPS-AnS-PdTPBP film (Fig. 3b) showed bright up-converted blue fluorescence in the air at room temperature (Fig. 3c). The upconversion fluorescence spectra showed an emission maximum at 478 nm with an anti-Stokes shift, Δ*E*_UC_ = 0.62 eV (Fig. 3d).^62^ The absence of normal emission maximum at 451 nm in up-conversion emission spectra confirmed the secondary inner filter effect. A significant quenching of PdTPBP phosphorescence at 798 nm confirmed triplet sensitization of TIPS-AnS by PdTPBP in the G-TXr-TIPS-AnS-PdTPBP films (Fig. 3d and S16†). The double logarithmic plots of excitation intensity dependency of up-conversion emission at 478 nm of G-TXr-TIPS-AnS-PdTPBP films with the change in slopes from 2 to 1 confirmed the sensitized TTA-UC mechanism (Fig. 3e).

A long triplet excited-state lifetime of annihilator, *τ*_T_ = 1.4, 1.5, and 1.7 ms in sample-III, sample-II, and sample-I, respectively, confirmed the protection of annihilator triplets against quenching by molecular oxygen (Fig. 3f and S17†). The *τ*_T_ was determined from the global fitting of excitation intensity-dependent up-conversion emission decay kinetics according to a previously developed method using eqn (1).^63–67^4
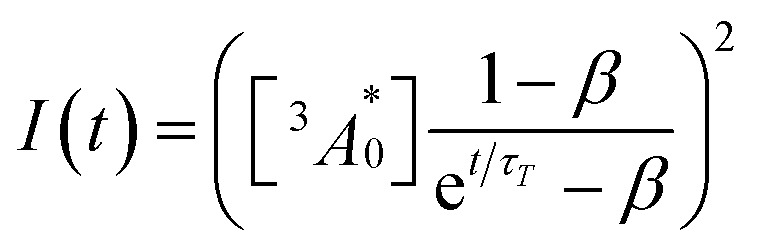
where *I*(*t*) is upconversion emission intensity, 
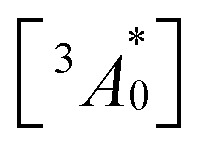
 is the concentration of triplet excited annihilators at time zero, and *β* is a dimensionless parameter describing the relative initial rate of triplet deactivation by TTA and spontaneous triplet decay.^68^ An increase in *β* with excitation intensity in sample-III confirmed a rise in annihilator triplet deactivation by TTA rather than spontaneous decay (Fig. S18†). A reference TTA-UC experiment in the aerated TXr-TIPS-AnS-PdTPBP solution (Fig. S19,†*λ*_ex_ = 633 nm laser, *I*_ex_ = 15 W cm^−2^) showing no upconversion emission confirmed the role played by gelatin in the protection of chromophore triplets against oxygen quenching in the film.

A maximum internal UC quantum yield, *Φ*_UC_ = 8.5% (50% theoretical maximum) was observed in sample-III. The *Φ*_UC_ was calculated by a relative method using cresyl violet perchlorate (CV)^55^ as a reference standard in G-TXr-TIPS-AnS-CV film (see methods section for detailed procedure). The internal *Φ*_UC_ was calculated by reabsorption correction using normalized emission spectra of TIPS-AnS in TXr in the absence of a sensitizer (Fig. S6†). Without reabsorption correction, a maximum external *Φ*_UC_ = 7.3% was observed in sample-III (Fig. S20†). The *Φ*_UC_ = 8.5% is comparable to the record value for red to blue TTA-UC, *Φ*_UC_ = 8.2% in deaerated nanocellulose film^29^ and is higher than *Φ*_UC_ = 5.6% reported in deaerated PdTPBP/DPA crystals.^28^

The high *Φ*_UC_ observed for sample-III in the air can also be accounted for high PdTPBP-to-TIPS-AnS triplet energy transfer (*Φ*_TET_ = 94%). The *Φ*_TET_ was calculated from phosphorescence lifetimes in the presence (*τ*_P_) and absence (*τ*_Po_) of TIPS-AnS in the G-TXr-PdTPBP film (Fig. S21†) using eqn (2).5
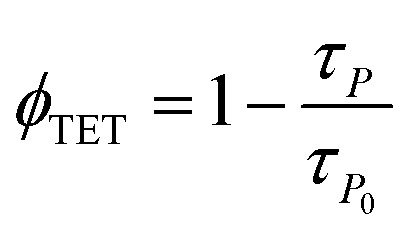


Interestingly, *Φ*_UC_ decreased upon increasing the concentration of TIPS-AnS from sample-III to sample-I even after correction for secondary inner filter effects. It can be due to the nanoaggregation of the excited state of TIPS-AnS which could not be detected in UV-vis or emission spectra of sample-I to sample-III (Fig. S22 and S15†). To further ascertain the concertation effect of TIPS-AnS, we carried out TTA-UC experiments at even higher concentrations of TIPS-AnS (25 mmol kg^−1^) in the G-TXr-TIPS-AnS-PdTPBP film denoted as sample-X. Interestingly we did not observe any UC emission in the sample-X. The emission spectrum of TIPS-AnS in sample-X is slightly red-shifted compared to that in sample-I (Fig. S23†). Additionally, sample-X showed a low apparent *Φ*_f_ = 6.2%, compared to *Φ*_f_ = 79% in the film with 200 μmol kg^−1^ of TIPS-AnS (measured by an absolute method using an integration sphere). Together these results indicate that the excited state of TIPS-AnS is quenched at high concentrations, possibly due to the aggregation or structural changes in the film.

The protection against photodegradation of chromophores in the G-TXr-TIPS-AnS-PdTPBP film was confirmed from stable UC emission upon continuous excitation with a 633 nm laser for 5600 s (Fig. S24†). One mechanism of photodegradation is a reaction between the chromophore and singlet oxygen, where the singlet oxygen is generated by triplet sensitization from a chromophore. Stable UC emission in deaerated TXr-TIPS-AnS-PdTPBP solution upon continuous irradiation at high laser excitation intensity of 15.5 W cm^−2^ (Fig. S25†) confirmed that the sensitizer and annihilator are stable against photodegradation in absence of oxygen. However, the TXr-TIPS-AnS-PdTPBP films showed a minor decrease in the UC signal upon continuous excitation for 1800 s (Fig. S26†) and the UC stability time decreased with higher excitation intensity. This can be due to minor photodegradation of the chromophores caused by the reaction with residual O_2_ in the film. It was confirmed by a slight decrease in absorbance of chromophores after TTA-UC measurements (Fig. S27†).

Further, to understand the TTA-UC operation inside the trapped TXr liquid in the film, we measured UC properties in deaerated TXr-TIPS-AnS-PdTPBP solution with [PdTPBP] = 5 μM and [TIPS-AnS] = 1 mM, these are the same initial concentrations of chromophores used to prepare sample-I before casting. The deaerated TXr-TIPS-AnS-PdTPBP solution showed an increase in UC emission with an increase in excitation intensity (Fig. S28a†). The *I*_th_ = 415 mW cm^−2^ and *Φ*_UC_ = 5.7% observed in the TXr-TIPS-AnS-PdTPBP solution is comparable to that observed for film sample-I (Fig. S28b and c†). The lower *I*_th_ observed in the film could be due to a five-fold increase in sensitizer concentration compared to degassed TXr liquid.

### Generalization of the concept to far-red to blue TTA-UC

The versatility of the developed strategy is generalized for far-red to blue TTA-UC by paring an S-T sensitizer, Os(*m*-peptpy)_2_(TFSI)_2_ recently published by us (Sasaki *et al.*,^51^Fig. 1b) with TIPS-AnS in a G-TXr-TIPS-AnS-Os(*m*-peptpy)_2_(TFSI)_2_ film (Fig. 4a, see methods for detailed preparation procedure). The S-T sensitizers are metal–organic complex molecules that show direct absorption of ground-state singlet to excited triplet state in the far-red/NIR region. These sensitizers are useful to avoid the energy losses during the inter-system crossing in a sensitized TTA-UC process.^51^ The Os(*m*-peptpy)_2_(TFSI)_2_ shows a long phosphorescence lifetime, *τ*_p_ = 80 μs due to the perylene units at the *meta* position acting as a triplet reservoir, which minimizes the heavy atom effect of Osmium.^51^ The absorption spectra of Os(*m*-peptpy)_2_(TFSI)_2_ show vibronic peaks at 424, 450, 495, and 673 nm whereas emission spectra of Os(*m*-peptpy)_2_(TFSI)_2_ show triplet metal to ligand charge transfer (^3^MLCT) emission at 753 nm (1.65 eV) and phosphorescence emission from the perylene unit (^3^pPe) at 822 nm (1.51 eV, *λ*_ex_ = 640 nm, Fig. S29†).^51^

In the G-TXr-TIPS-AnS-Os(*m*-peptpy)_2_(TFSI)_2_ film, Os(*m*-peptpy)_2_(TFSI)_2_ shows absorption peaks at 496 and 674 nm with a tail ending in the far-red region (inset Fig. 4c), whereas ^3^MLCT emission was observed at 743 nm (1.67 eV) and ^3^pPe at 820 nm (1.51 eV) upon *λ*_ex_ = 640 nm excitation. The triplet energy of Os(*m*-peptpy)_2_(TFSI)_2_ at 1.51 eV is suitable for exergonic triplet sensitization of TIPS-AnS (*T*_1_ ≈ 1.4 eV).^47,48^ The G-TXr-TIPS-AnS-Os(*m*-peptpy)_2_(TFSI)_2_ film showed blue UC emission, observed with naked eyes upon excitation with 700 nm, nanosecond pulsed laser, *I*_ex_ = 0.9 mJ pulse^−1^ (Fig. 4b). The UC emission was further confirmed from the rise and decay in emission signals at *λ*_em_ = 478 nm, upon excitation with 700 nm and 730 nm far-red nanosecond pulsed laser excitation with an anti-stokes shift, Δ*E*_UC_ = 0.752 eV (Fig. 4d).^62^ Hence, through the successful execution of this work, we have now expanded the photon harvesting spectral range of TTA-UC bioplastics from 500 to 730 nm.^46^

### Recycling of the chromophores from the G-TXr-TIPS-AnS-PdTPBP bioplastics

**Fig. 5:**
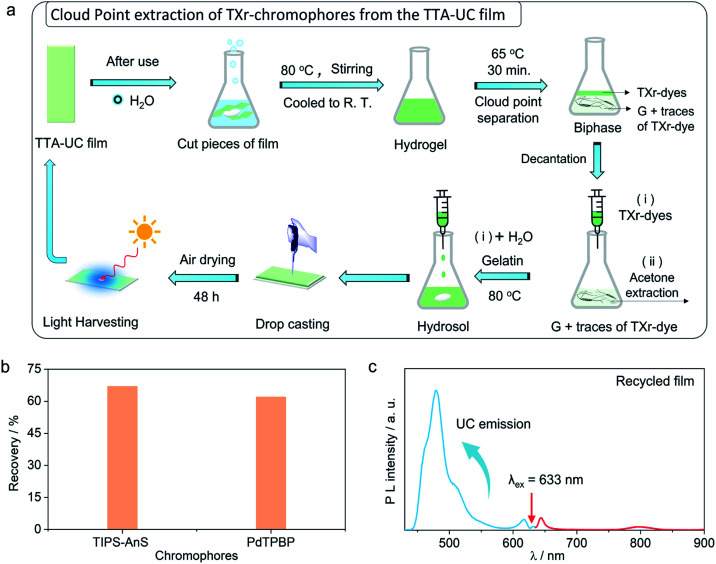
Circular use of key bioplastics components. (a) Illustration of the recycling of G-TXr-TIPS-AnS-PdTPBP film. (b) Plot showing % recovery of chromophores and (c) UC emission spectrum of the recycled film upon 633 nm laser excitation (*I*_ex_ = 1.3 W cm^−2^).

The use of petroleum-based plastics in photonics is attractive due to their tunable molding, mechanical flexibility, and optical transparency.^69^ However, non-biodegradation of most of them, and non-recyclability of toxic chromophores fabricated inside them renders them unattractive from a sustainability perspective.^37,38^ To avoid the similar fate of our TTA-UC bioplastics we developed a new downstream recycling approach of “cloud point extraction of TXr-chromophores” from the waste G-TXr-TIPS-AnS-PdTPBP bioplastics (Fig. 5a). The cloud point of a nonionic surfactant is the temperature at which it begins to phase separate from the solid due to incomplete dissolution.^70^ The TXr has a cloud point of 65 °C in water^71^ and hence underwent phase-separation from the G-TXr-TIPS-AnS-PdTPBP hydrogel when kept at 65 °C for 30 min (Fig. 5a and S30†). The phase-separated TXr-chromophores top layer was extracted using a syringe. The remaining TXr-chromophores mixture in the gelatin phase was re-extracted using acetone as antisolvent (see methods for detailed extraction procedure). The re-extracted TXr-chromophores mixtures were dissolved in 1 mL of water separately to record their absorption spectra to calculate % chromophores extraction (Fig. S31†).

Overall, 67% (55% + 12%) of [TIPS-AnS] and 62% (56% + 6%) of [PdTPBP] could be re-extracted using cloud point and acetone extraction (Fig. 5b). The re-extracted TXr-chromophores mixture was used to prepare G-TXr-TIPS-AnS-PdTPBP film by dissolving in fresh gelatin (Fig. 5a and S30†). The air-dried film showed blue upconversion emission upon excitation with a 633 nm laser in the air (Fig. 5c). The waste gelatin can be discarded for biodegradation after proper washing.

## Conclusions

This work establishes TTA-UC bioplastics as recyclable solid-state upconversion materials for broad-spectrum solar light harvesting. The results demonstrate our idea of realizing sustainable and efficient solid-state TTA-UC materials by encapsulating TTA-UC chromophores liquid inside the biopolymer film for efficient channelization of triplet energy in the air. Moreover, it also established the necessity of an ionic annihilator to fabricate transparent TTA-UC bioplastic films made of gelatin with TXr as chromophore dispersing surfactant. The TTA-UC bioplastics are very easy to prepare in an energy-efficient ambient condition without any complicated procedures previously reported to prepare solid-state TTA-UC materials of petroleum-based polymers. Moreover, the simple recycling approach developed in this work to re-extract the expensive and toxic chromophores give them a significant advantage over existing solid-state TTA-UC materials of petroleum-based polymers on grounds of sustainability.

In conclusion, we have developed new G-TXr-TIPS-AnS-PdTPBP and G-TXr-TIPS-AnS-Os(*m*-peptpy)_2_(TFSI)_2_ bioplastic films to upconvert red and far-red light to blue light. To realize this, we synthesized a new ionic annihilator, sodium-TIPS-anthracene-2-sulfonate (TIPS-AnS), with suitable triplet energy to pair with PdTPBP/Os(*m*-peptpy)_2_(TFSI)_2_ triplet sensitizers and consequent fabrication into photon upconverting bioplastics. Chromophores in the bioplastics diffuse mainly in the dispersed TXr liquid phase for efficient triplet energy transfer, followed by efficient triplet–triplet annihilation due to the oxygen protection provided by thick gelatin fiber networks. Consequently, the developed red to blue G-TXr-TIPS-AnS-PdTPBP bioplastics showed a record UC quantum yield, *Φ*_UC_ = 8.5% in the solid-state in the air with an anti-Stokes shift of 0.62 eV. The developed strategy was generalized for far-red to blue TTA-UC in the G-TXr-TIPS-AnS-Os(*m*-peptpy)_2_(TFSI)_2_ film with an anti-Stokes shift of 0.75 eV upon 730 nm far-red laser excitation, thus expand the photon harvesting window of TTA-UC bioplastics from 500–730 nm.^44^ Finally, we developed a simple and effective recycling route of “cloud point extraction of chromophores” from G-TXr-TIPS-AnS-PdTPBP bioplastic film, demonstrating circular use of key functional components and illustrating how to avoid future post-utilization leaching of chromophores into the environment. Using this route, we could recycle 67% of TIPS-AnS and 62% of PdTPBP along with TXr. The recycled TXr-chromophores mixture was successfully re-used to fabricate new TTA-UC bioplastics thus establishing circularity of the developed bioplastics platforms. The developed strategies offers a new direction to design recyclable optical bioplastics materials in energy harvesting applications to strengthen the concept of circular bioeconomy.

## Author contributions

P. B. and K. M. P. conceived this idea and obtained financial support for the work. P. B. led the experimental work assisted by F. E., H. B., Y. S., S. G., A. M. Kimizuka lab (Y. S., N. Y., and N. K.) provided the far-red/NIR sensitizer. F. E., K. B., B. A., and K. M. P. assisted P. B. in data analysis. P. B. and K. M. P. wrote the first draft of the manuscript. All authors participated in interpretation of the results and provided input on the manuscript.

## Conflicts of interest

There are no conflicts to declare.

## Supplementary Material

TA-010-D2TA04810H-s001
