# Supplementary material for: Recyclable optical bioplastics platform for solid state red light harvesting via triplet–triplet annihilation photon upconversion
Source: J Mater Chem A Mater. 2022 Aug 16;10(40):21279–90. doi: 10.1039/d2ta04810h (PMC9578683; doi:10.1039/d2ta04810h)
Supplement: TA-010-D2TA04810H-s001 [file TA-010-D2TA04810H-s001.pdf]

Electronic Supplementary Material (ESI) for Journal of Materials Chemistry A.
This journal is © The Royal Society of Chemistry 2022

Supplementary Information

Recyclable Optical Bioplastics Platform for Solid State Red Light Harvesting via Triplet-triplet Annihilation Photon Upconversion

Pankaj Bharmoria,[a] Fredrik Edhborg,[a] Hakan Bildirir,[a] Yoichi Sasaki,[b] Shima Ghasemi, [a] Anders Mårtensson,[a] Nobuhiro Yanai,[b] Nobuo Kimizuka,[b] Bo Albinsson,[a] Karl Börjesson,[c] Kasper Moth-Poulsen*[a,d,e]

[a.] Department of Chemistry and Chemical Engineering, Chalmers University of Technology, Kemivägen 4,412 96, Gothenburg, Sweden.

[b.] Department of Chemistry and Biochemistry, Graduate School of Engineering, Center for Molecular Systems (CMS), Kyushu University 744 Moto-oka, Nishi-ku, Fukuoka 819-0395, Japan

[c.] Department of Chemistry and Molecular Biology, Kemivägen 10, 412 96, Gothenburg, Sweden

[d.] The Institute of Materials Science of Barcelona, ICMAB-CSIC, 08193, Bellaterra, Barcelona, Spain

[e.] Catalan Institution for Research & Advanced Studies, ICREA, Pg. Lluís Companys 23, Barcelona, Spain

*To whom correspondence should be addressed: E-mail: pankajb@chalmers.se, kasper.moth-poulsen@chalmers.se

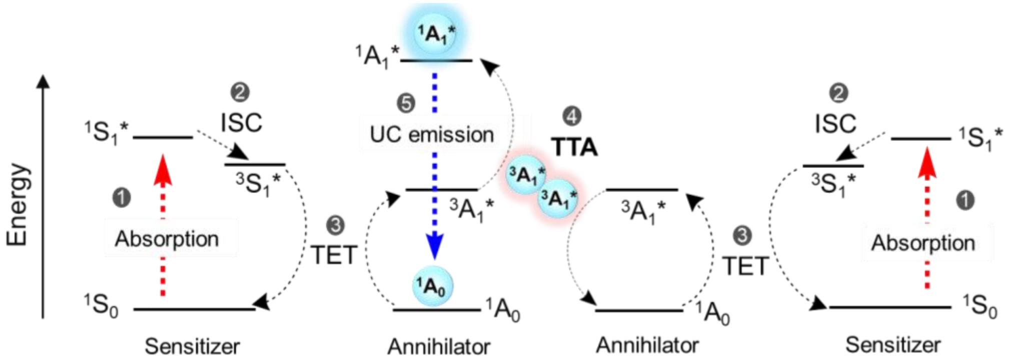

Fig. S1. Energy diagram of triple-triplet annihilation photon upconversion (TTA-UC).

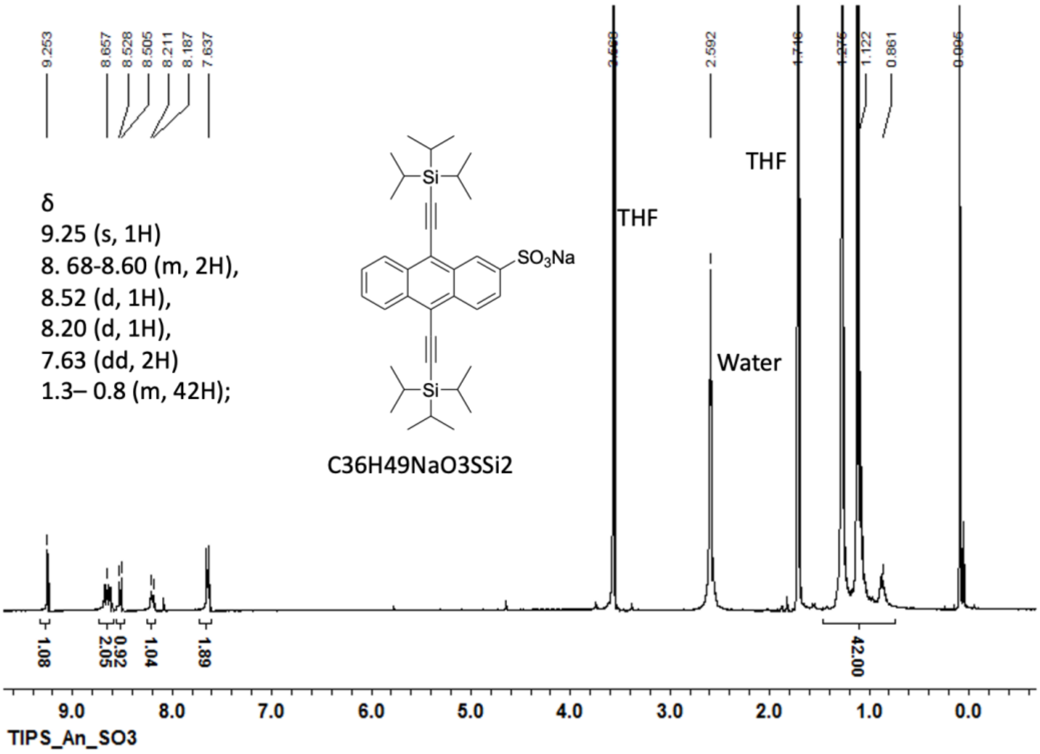

Fig. S2. 1H-NMR Spectra of Sodium TIPS-Anthracene Sulfonate in deuterated THF. ^{1}H NMR (400 MHz, d^8-THF)

a

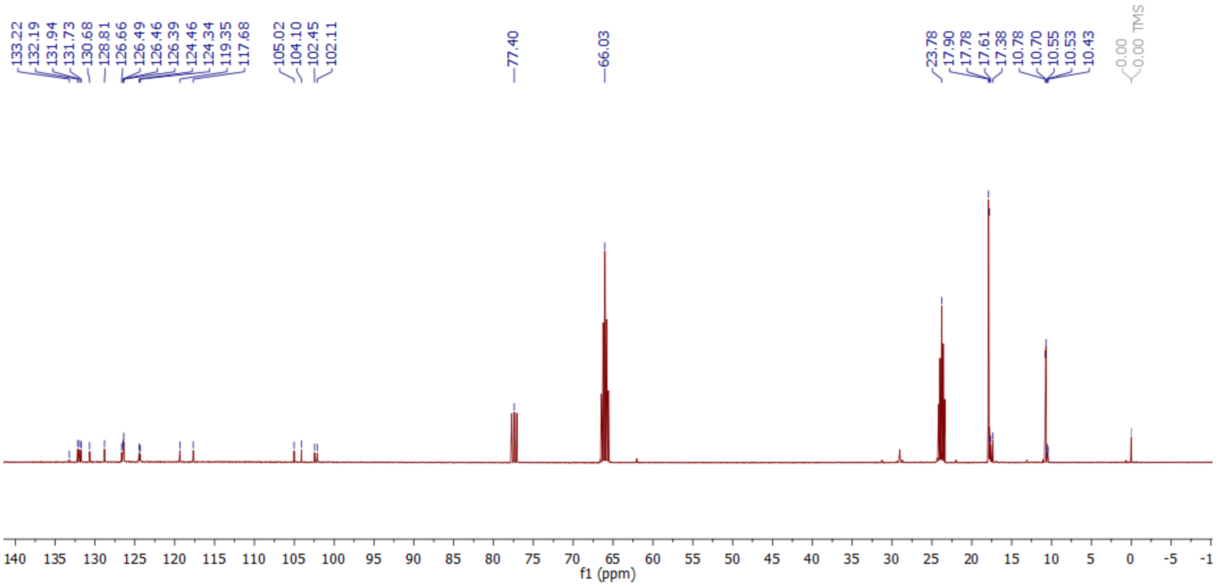
133.22
132.19
131.94
131.73
130.68
128.81
126.66
126.49
126.46
126.39
124.46
124.34
119.35
117.68
105.02
104.10
102.45
102.11
77.40
66.03
23.78
17.90
17.78
17.61
17.38
10.78
10.70
10.55
10.53
10.43
0.00
0.00 TMS
f1 (ppm)

b

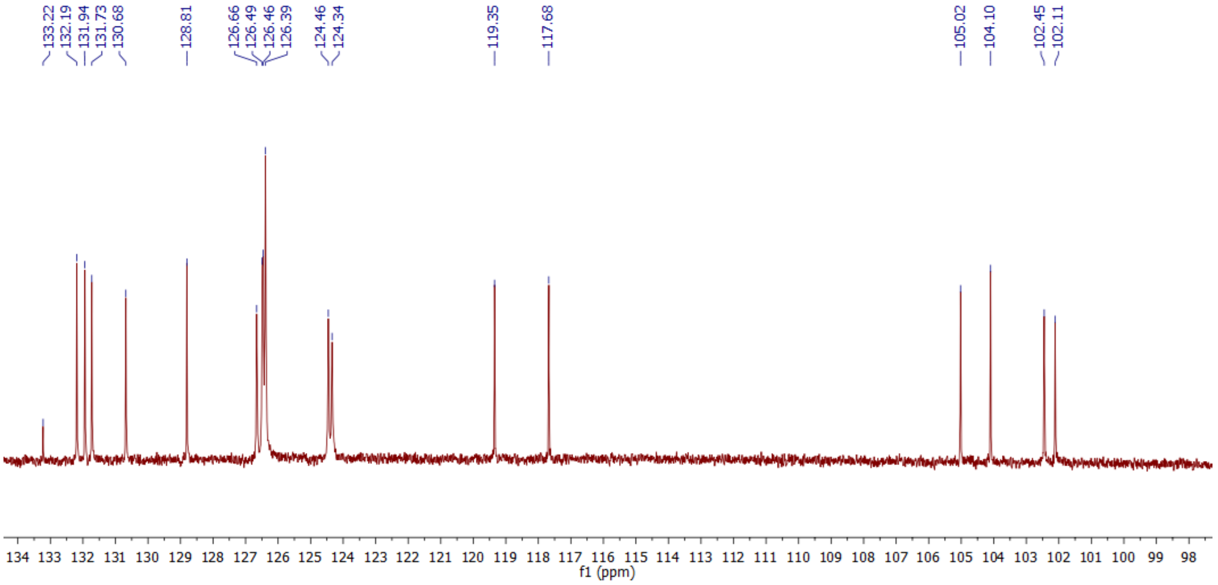
133.22
132.19
131.94
131.73
130.68
128.81
126.66
126.49
126.46
126.39
124.46
124.34
119.35
117.68
105.02
104.10
102.45
102.11
f1 (ppm)

c

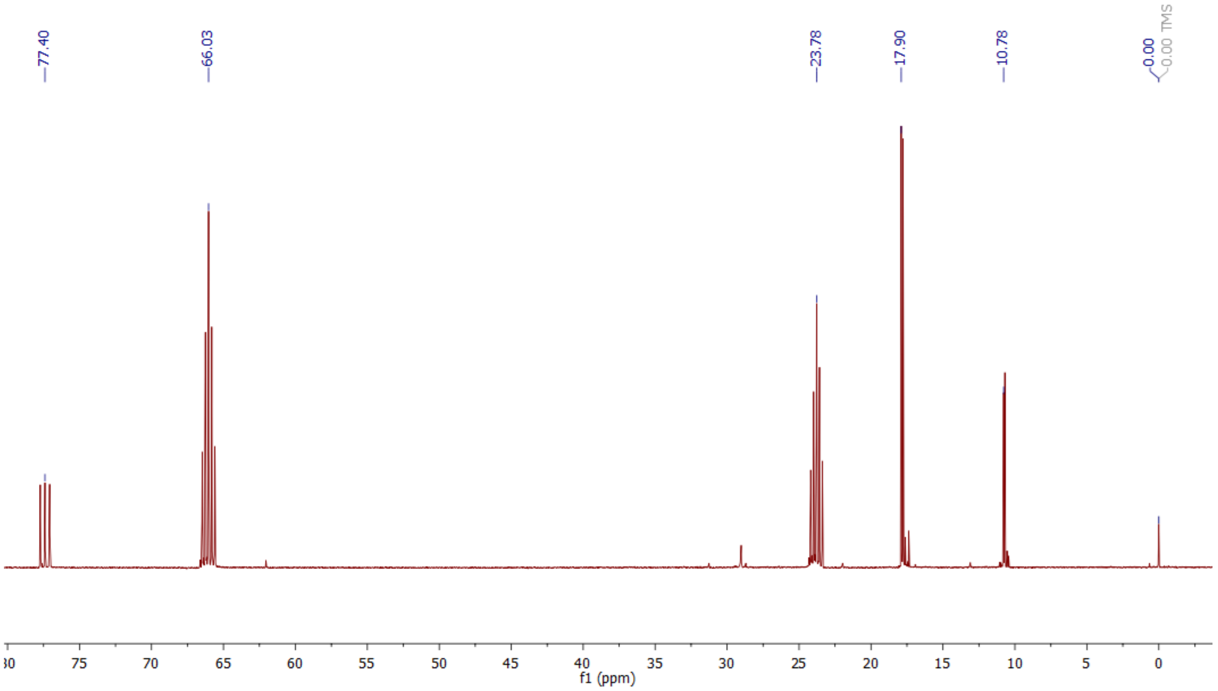

Fig. S3. ^{13}C carbon NMR of TIPS-AnS in $CDCl_3$ and d-THF mixture. **a)** Full spectrum **b**) sp2 and sp carbons, **c)** aliphatic region (77.40 comes from chloroform and 66.03 & 23.78 are from THF).

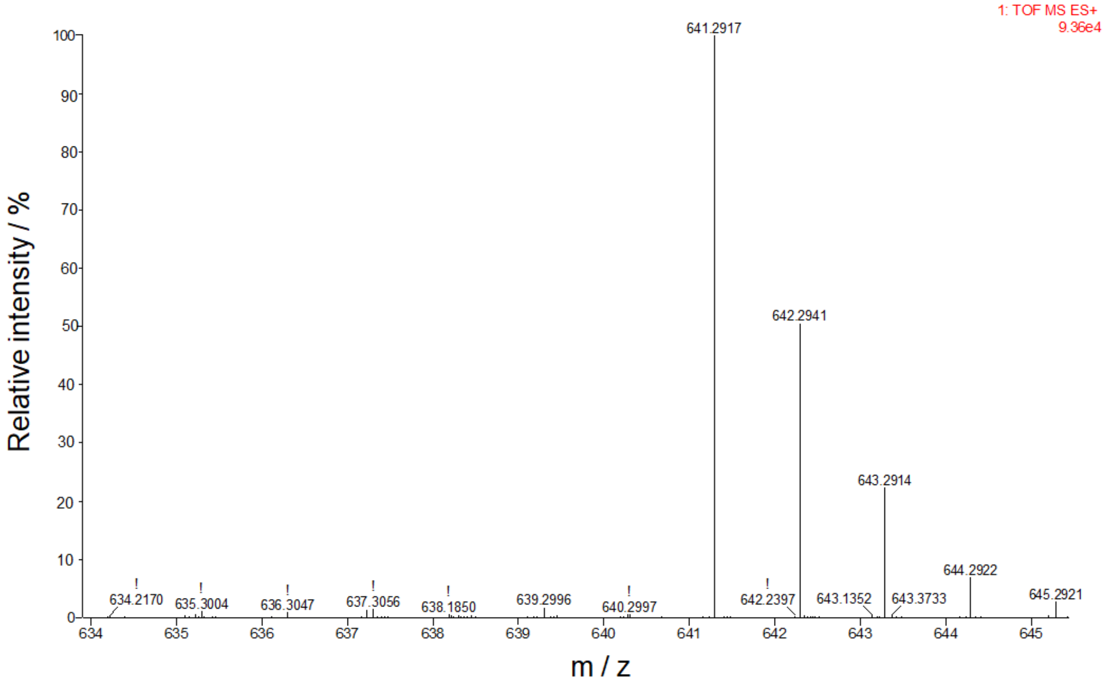

Fig. S4. HR-MS spectra of TIPS-Anthracene sodium-2-sulfonate

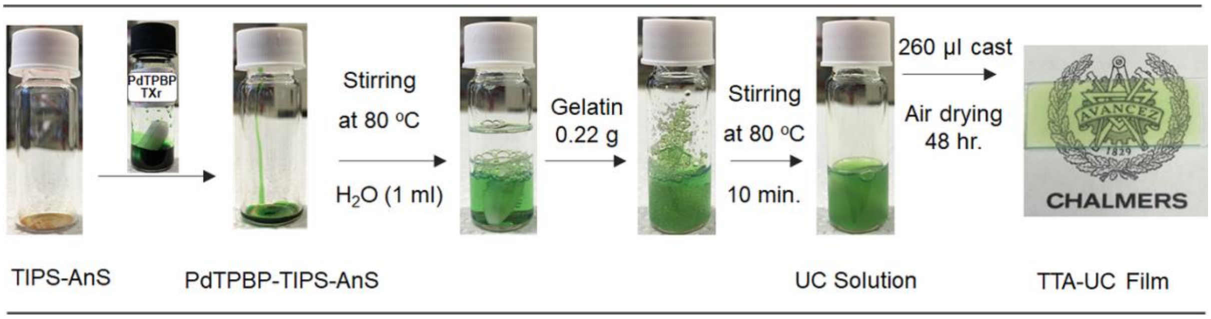

Fig. S5. Schematic of the preparation of G-TXr-TIPS-AnS-PdTPBP film (Sample-I).

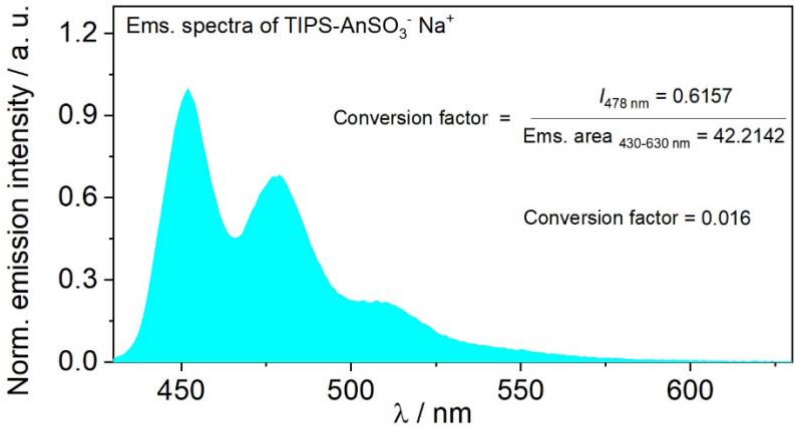

Fig. S6. Fluorescence emission spectra of TIPS-AnS (40 µM) in TXr liquid in air (Inset shows correction factor used for calculation of internal UC quantum yield of G-TXr-TIPS-AnS-PdTPBP films using UC emission intensities at 478 nm).

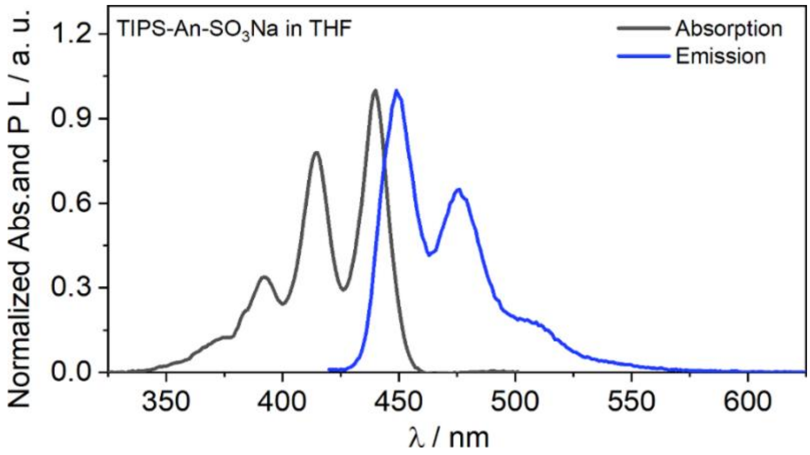

Fig. S7. Normalized absorption (black line) and photoluminescence (PL, blue lines) spectra of the TIPS-AnS (blue line, λ_{ex} = 415 nm) in THF. Chromophore concentrations (TIPS-AnS = 40 µM).

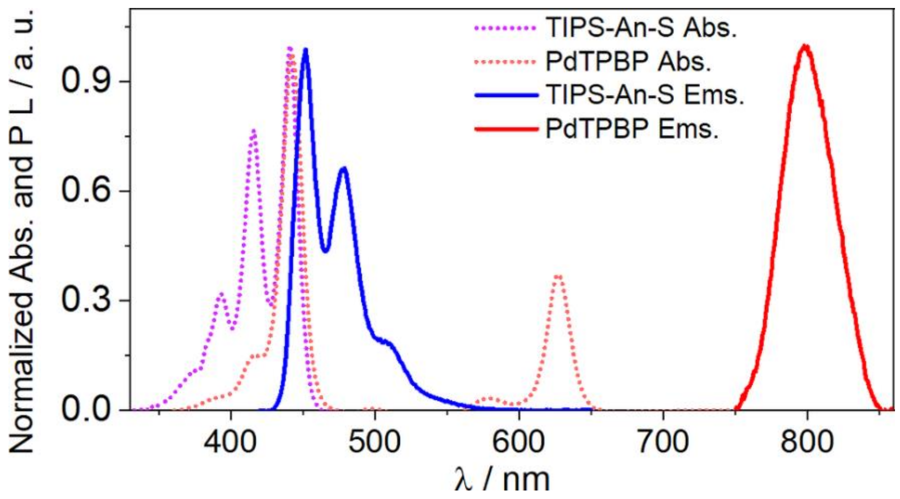

Fig. S8. Normalized absorption (dotted lines) and photoluminescence (PL, solid lines) spectra of the TXr-TIPS-AnS (blue line, λ_e = 415 nm) and TXr-PdTPBP (red line, λ_{ex} = 633 nm) in air. Chromophore concentrations TIPS-AnS = 40 µM and PdTPBP = 5 µM.

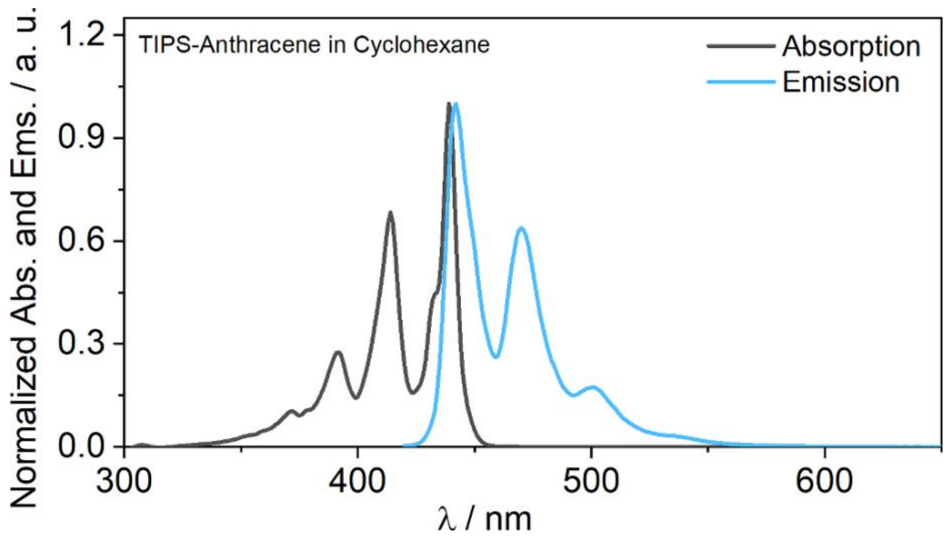

Fig. S9. Normalized absorption (black line) and photoluminescence (PL, blue lines) spectra of the TIPS-Anthracene (blue line, λ_{ex} = 414 nm) in cyclohexane. Chromophore concentrations (TIPS-AnS = 40 µM). The peaks were observed at λ_{abs} = 392nm, 414nm and 439 nm. λ_{ems} = 442 nm, 476nm and 501 nm.

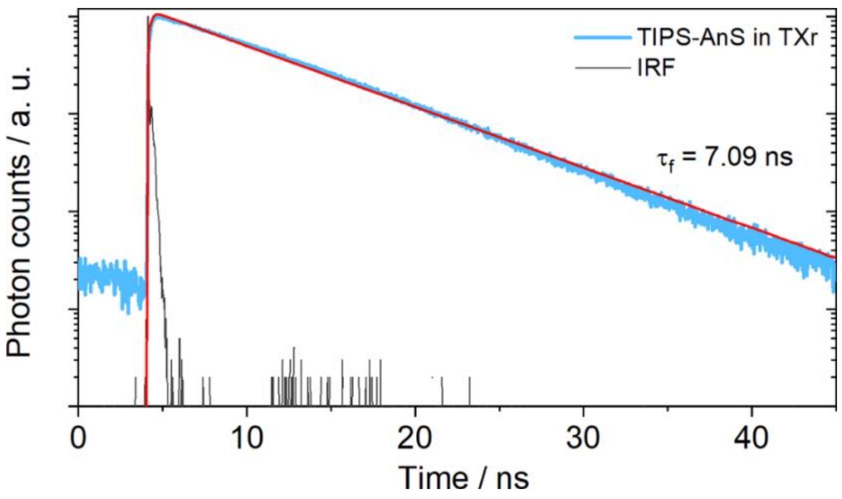

Fig. S10. Fluorescence decay profile of TIPS-AnS in TXr. Chromophore concentration TIPS-AnS=40 µM.

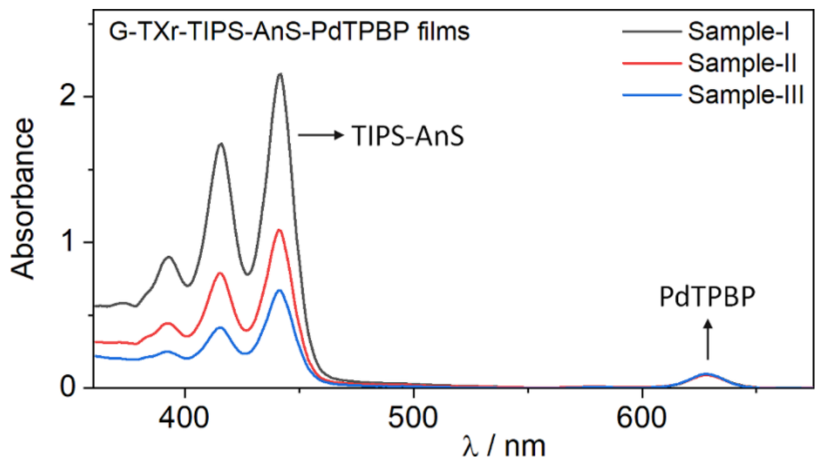

Fig. S11. Full absorption spectra of G-TXr-TIPS-AnS-PdTPBP films. TIPS-AnS concentrations (Sample-I = 5mmol.kg^{-1}, Sample-II = 2.5 mmol kg^{-1} and Sample-III = 1 mmol kg^{-1}).

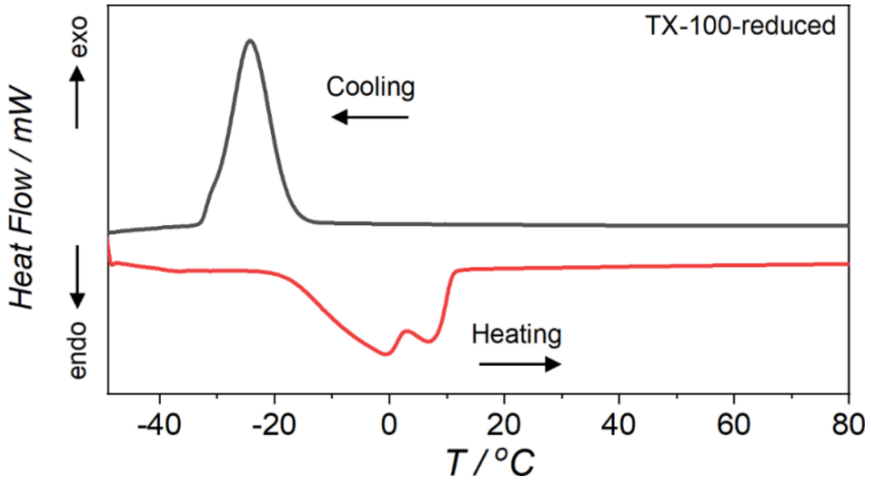

Fig. S12. DSC thermogram of vacuum dried TX-100-reduced. Scanning rate 10 °C min^{-1}.

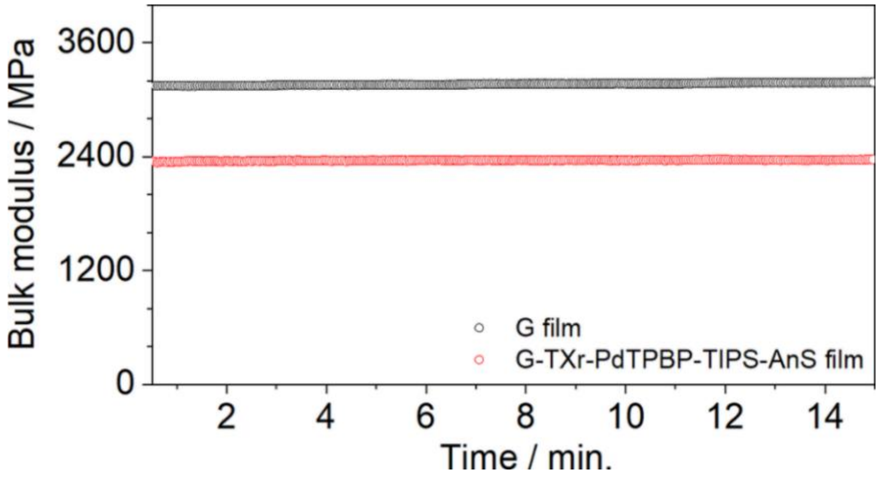

Fig. S13. Time sweeps dynamic mechanical analysis plots of the G film and the G-TXr-TIPS-AnS-PdTPBP film at 30 °C, a strain of 0.05 % and frequency of 1 Hz.

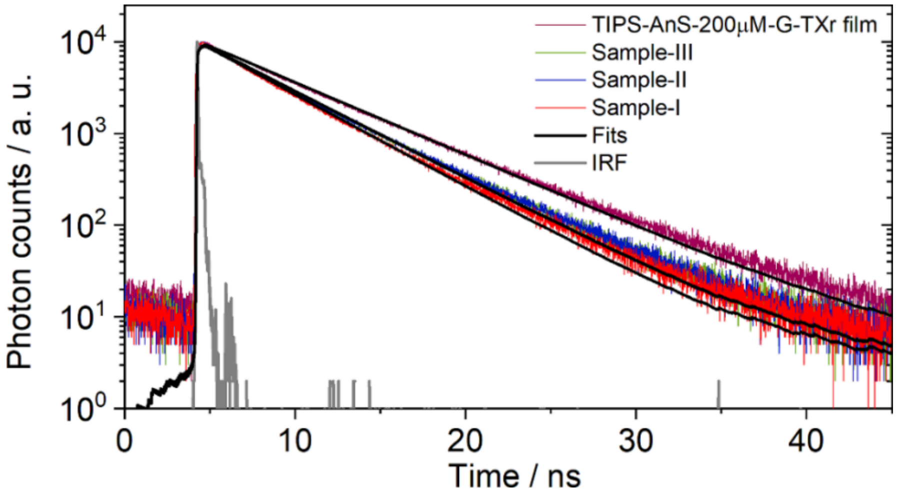

Fig. S14. Fluorescence decay profile of TIPS-AnS in the G-TXr-TIPS-AnS film or G-TXr-TXr-TIPS-AnS-PdTPBP films. TIPS-AnS concentrations (Sample-I = 5mmol kg^{-1}, Sample-II = 2.5 mmol kg^{-1} and Sample-III = 1 mmol kg^{-1}) and G-TXr-TIPS-AnS film (TIPS-AnS = 200 μmol kg^{-1}).

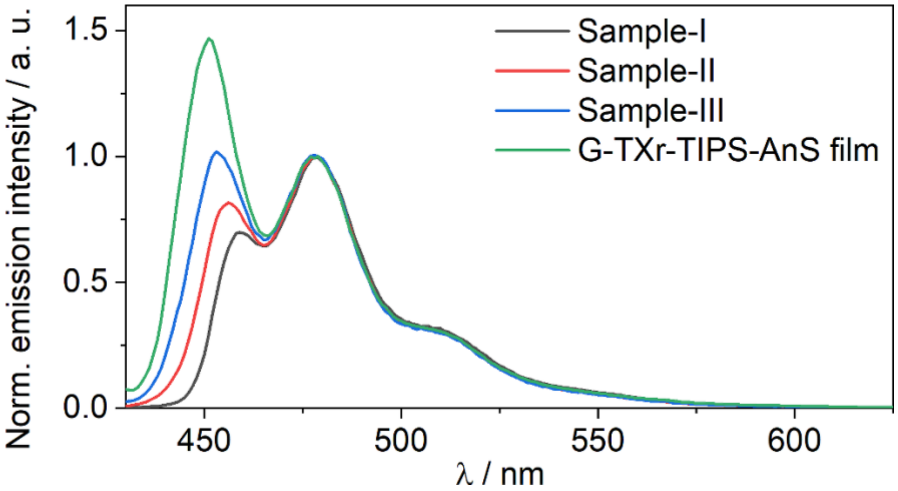

Fig. S15. Normalized emission spectra of TIPS-AnS in G-TXr-TIPS-AnS-PdTPBP films. TIPS-AnS concentrations (Sample-I = 5mmol kg^{-1}, Sample-II = 2.5 mmol kg^{-1} and Sample-III = 1 mmol kg^{-1}) and G-TXr-TIPS-AnS film (TIPS-AnS = 200 μmol kg^{-1}). The spectra are normalized corresponding to the wavelength at 478 nm.

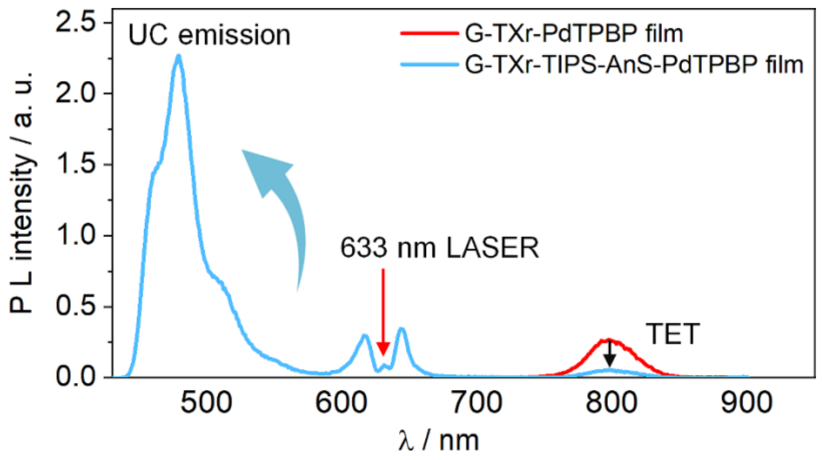

Fig. S16. Photoluminescence spectra of PdTPBP in the absence and presence of TIPS-AnS in sample-III. λ_{ex} = 633 nm, excitation intensity = 33.8 mW cm^{-2}.

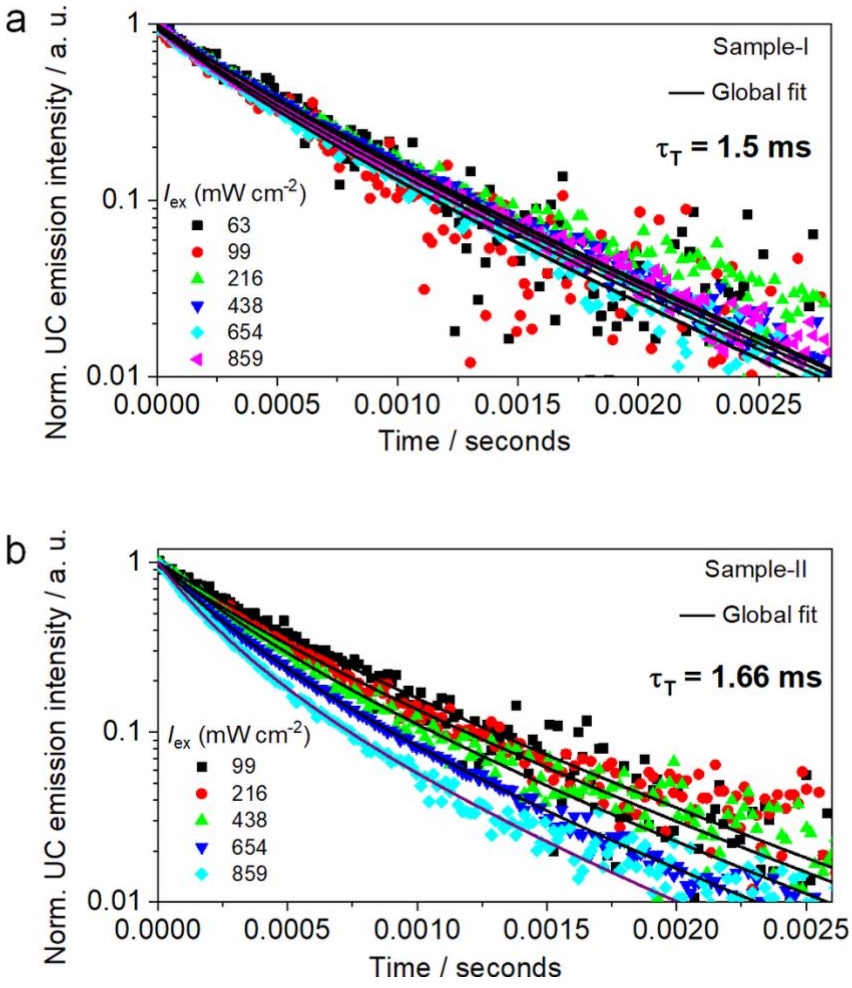

Fig. S17. Time resolved upconversion emission *vs* excitation intensity plot of G-TXr-TIPS-AnS-PdTPBP films in air. **a)** sample-I and **b)** sample-II.

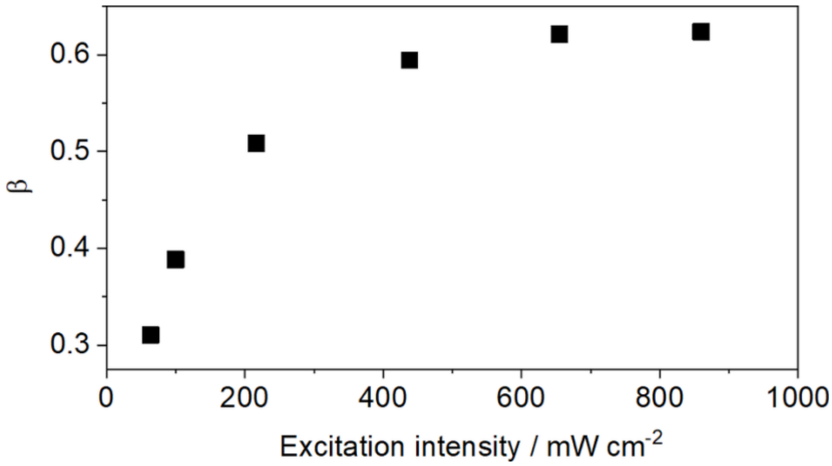

Fig. S18. Fitted value of β for the G-TXr-TIPS-AnS-PdTPBP Sample-III at various excitation intensities.

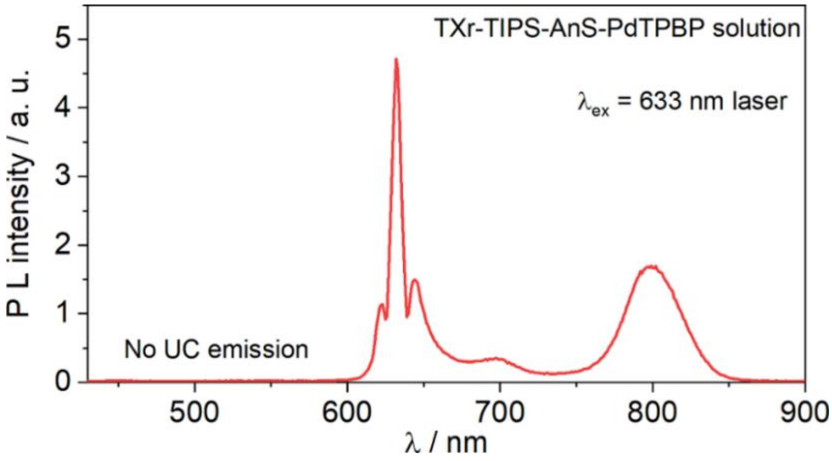

Fig. S19. Photoluminescence spectrum of the TXr-TIPS-AnS-PdTPBP solution at 633 nm laser (excitation intensity of 15 W cm^{-2}) in air.

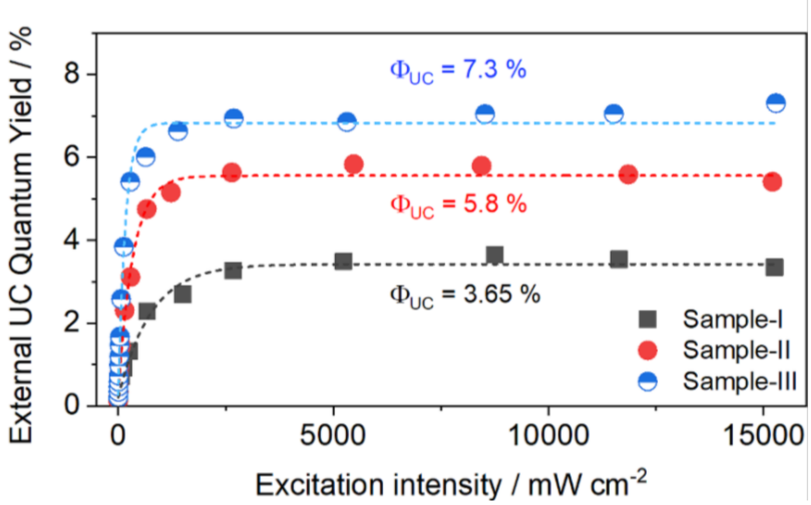

Fig. S20. Plot showing external UC quantum yields of G-TXr-TIPS-AnS-PdTPBP films calculated without conversion factor.

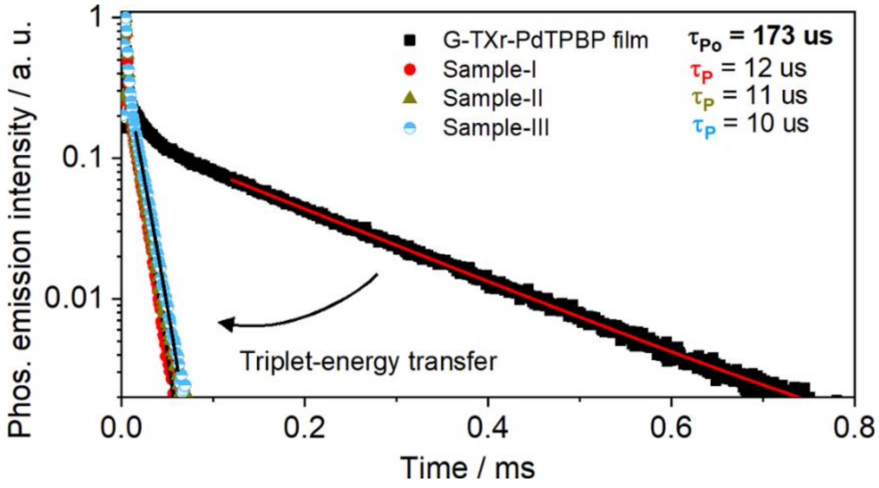

Fig. S21. Comparative phosphorescence decay profiles of G-TX-PdTPBP and TTA-UC film (Sample-I, Sample-II, and Sample-III).

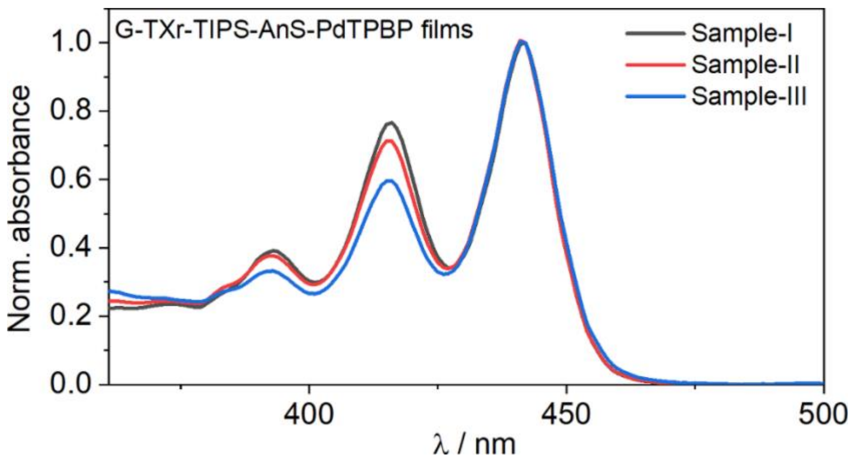

Fig. S22. Normalized absorption spectra of G-TXr-TIPS-AnS-PdTPBP films. TIPS-AnS concentrations (Sample-I = 5mmol kg^{-1}, Sample-II = 2.5 mmol.kg^{-1} and Sample-III = 1 mmol kg^{-1}).

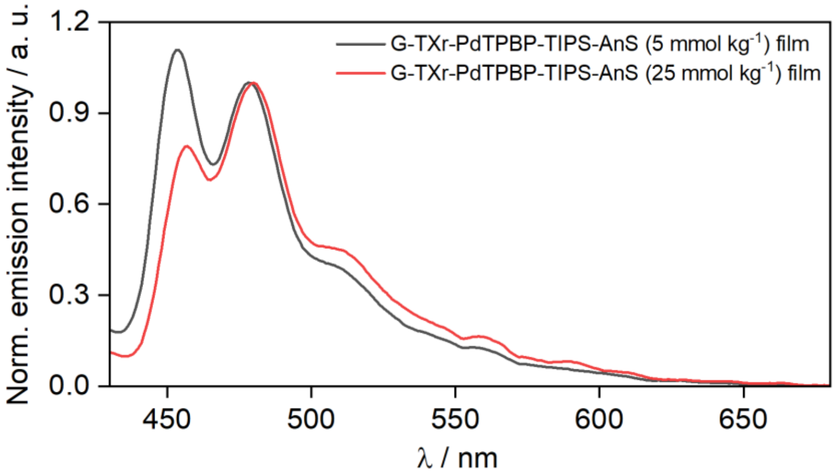

Fig. S23. Normalized emission spectra of G-TXr-TIPS-AnS-PdTPBP films. TIPS-AnS concentrations (TIPS-AnS = 5mmol kg^{-1} and TIPS-AnS = 25 mmol.kg^{-1}).

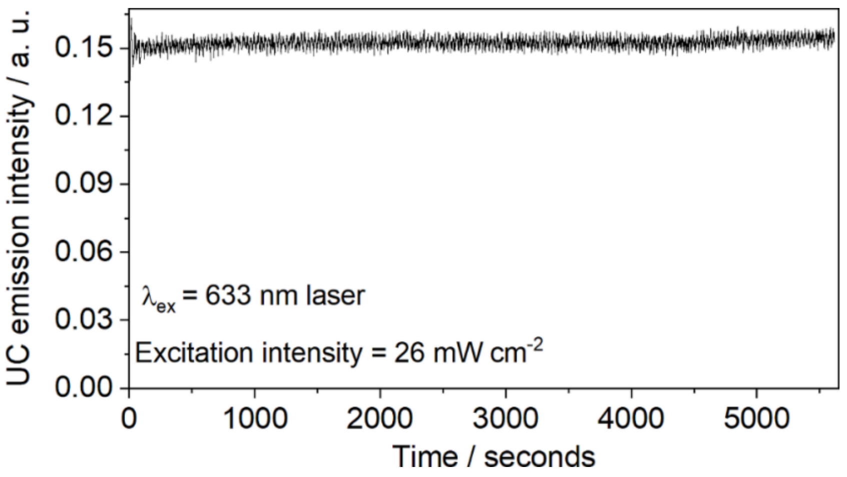

Fig. S24. UC emission intensity changes of the G-TXr-TIPS-AnS-PdTPBP film-I at λ_{em} = 478 nm upon continuous excitation with 633 nm laser for 5600 seconds (excitation intensity of 26 mW cm^{-2}) in air.

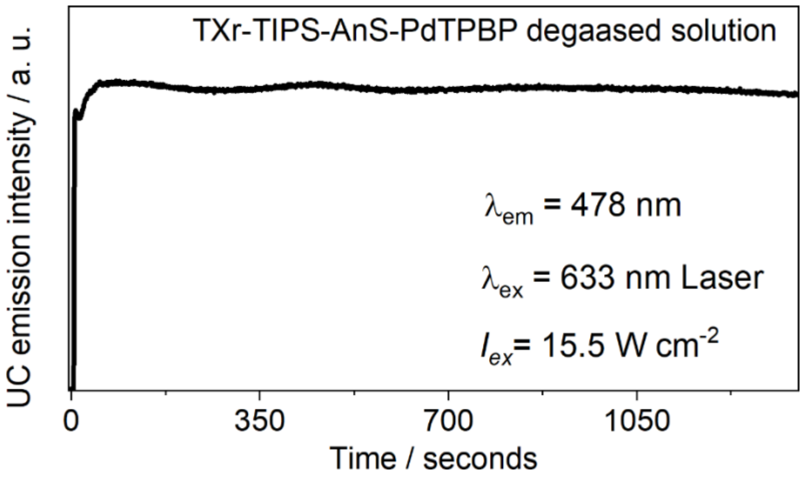

Fig. S25. Changes in upconversion emission intensity (λ_{em} = 478) of degassed TXr-TIPS-AnS-PdTPBP solution upon 633 nm laser excitation (excitation intensity of 15 W cm^{-2}).

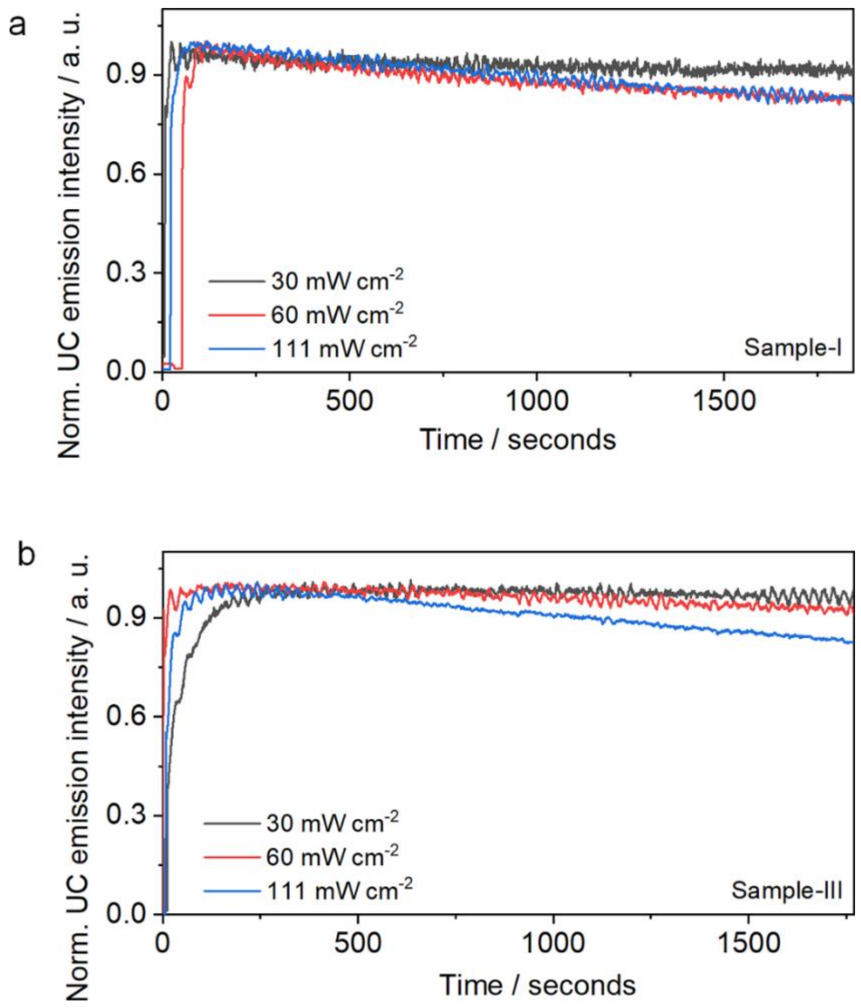

Fig. S26. UC emission intensity changes of the G-TXr-TIPS-AnS-PdTPBP film-I and film-III at λ_{em} = 478 nm upon continuous excitation with 633 nm laser for 1800 seconds at varying excitation intensities in air.

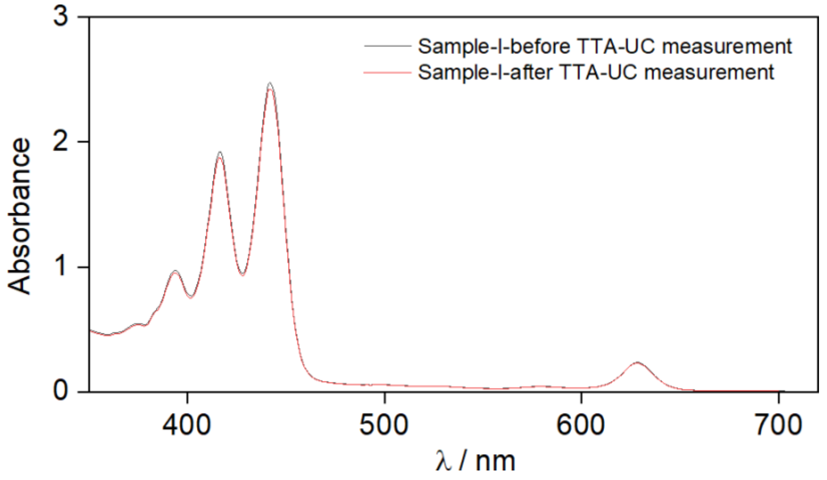

Figure S27. Absorption spectra of G-TXr-TIPS-AnS-PdTPBP film (Sample-I) before and after TTA-UC measurement.

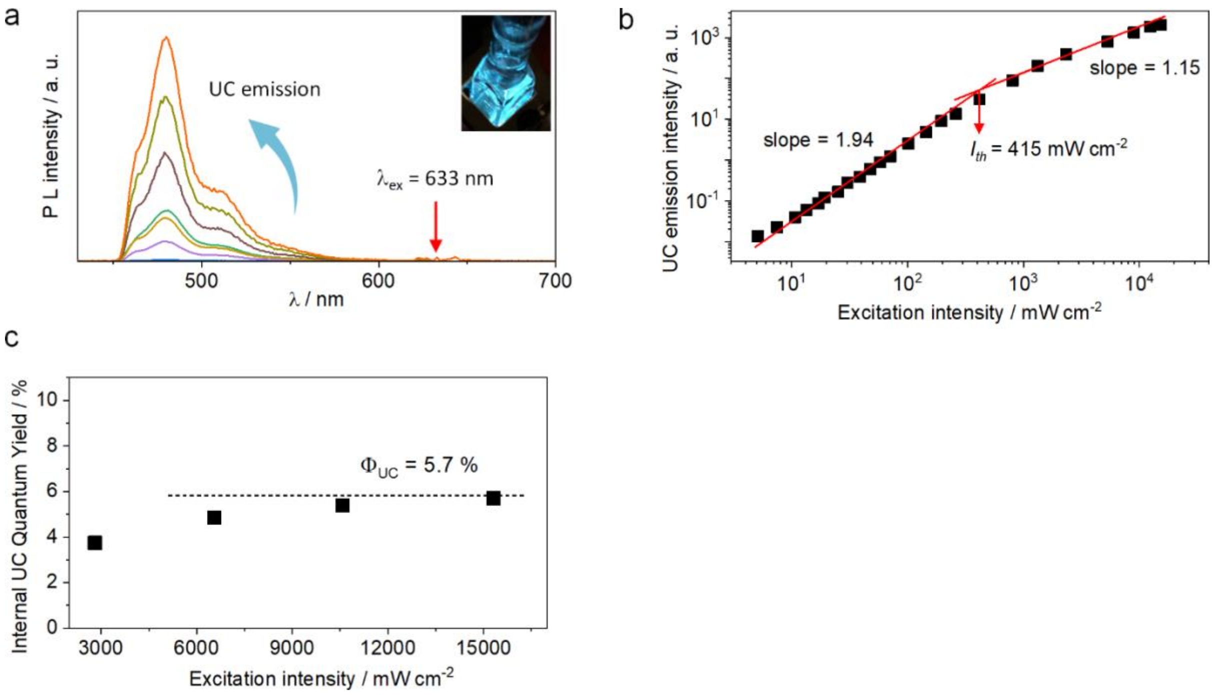

Fig. S28. Upconversion properties of deaerated TXr-TIPS-AnS-PdTPBP solution. **a)** PL spectra upon 633 nm laser excitation, inset show photograph of UC emission in the cuvette (no short pass filter used). **b)** Log–log plot of excitation intensity dependence of UC emission. **c)** Internal UC quantum yield *vs.* excitation intensity plot.

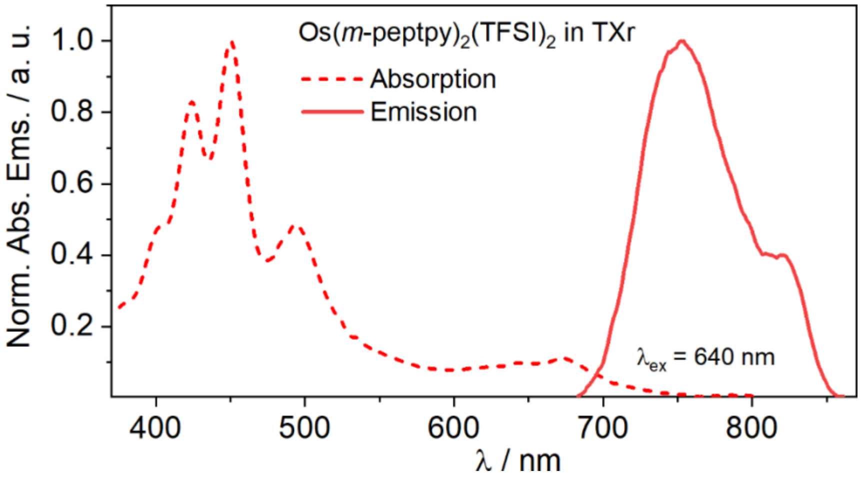

Fig. S29. Normalized absorption and emission spectra of Os(*m*-peptpy)$_2$(TFSI)$_2$ in TXr in air (λ_{ex} = 640 nm).

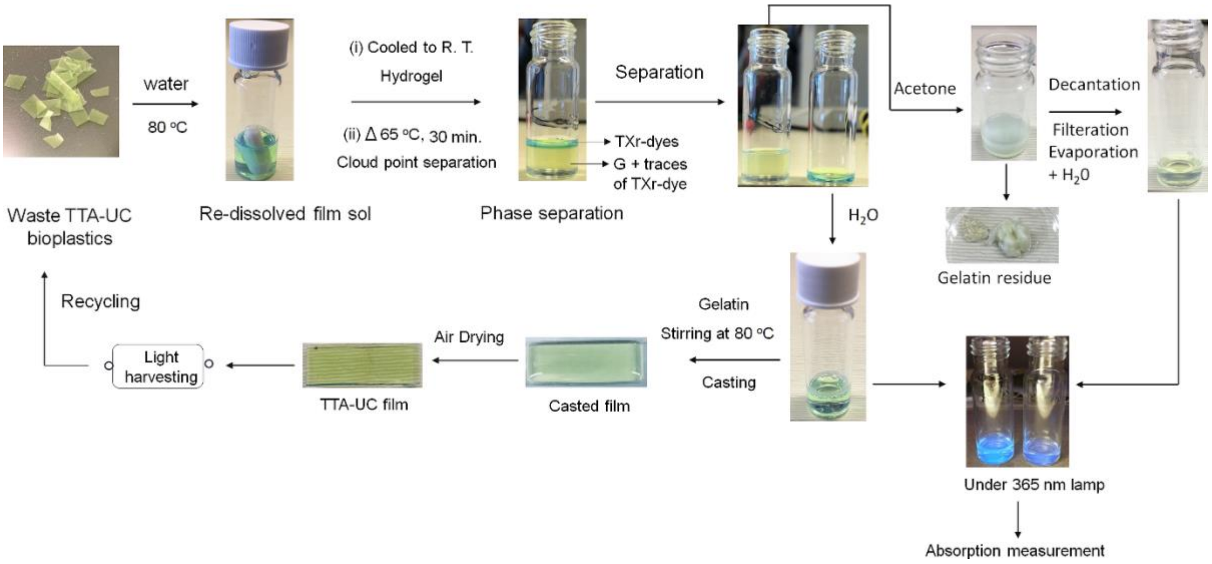

Fig. S30. Schematic of the recycling of G-TXr-TIPS-AnS-PdTPBP film.

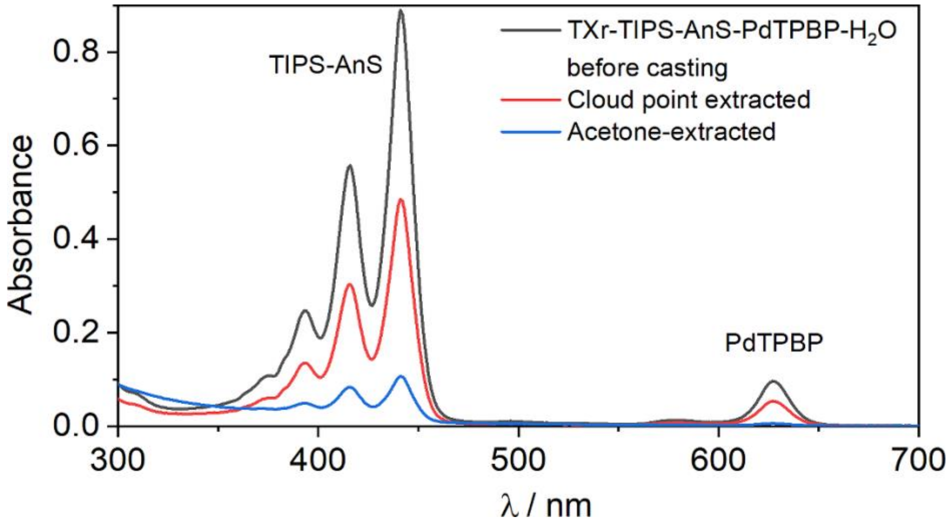

Fig. S31. Absorption spectra of TXr-TIPS-AnS-PdTPBP aqueous solution before casting into film and after cloud point and acetone extraction. The absorbance measurements carried out using 1 mm pathlength quartz cuvette.
